# Preventive application of lung growth factors and lack of attenuation of phenotype disruption of lung resident MSC from preterm infants by hyperoxia

**DOI:** 10.1186/s40348-026-00241-4

**Published:** 2026-05-22

**Authors:** Lena Holzfurtner, Judith Behnke, Pauline Korte, Maurizio J. Goetz, Jutta Petzinger, Anita C. Windhorst, Tayyab Shahzad, Stefano Rivetti, Ying Dong, Saverio Bellusci, Harald Ehrhardt

**Affiliations:** 1 Department of General Pediatrics and Neonatology, Justus-Liebig-University Giessen and Universities of Giessen and Marburg Lung Center (UGMLC), Member of the German Center for Lung Research (DZL), Giessen, 35392 Germany; 2 Institute of Medical Informatics (IMI), Justus-Liebig University Giessen, Giessen, 35392 Germany; 3 Justus-Liebig-University Giessen and Universities of Giessen and Marburg Lung Center (UGMLC), Excellence Cluster Cardio Pulmonary Institute (CPI), Member of the German Center for Lung Research (DZL), Giessen, 35392 Germany; 4 Department of Neonatology, Charité – Universitätsmedizin Berlin, Berlin, 13353 Germany; 5 Institute for Lung Health (ILH), Giessen, 35392 Germany; 6 German Centre for Child and Adolescent Health (DZKJ), Partner Site Berlin, Berlin, Germany

**Keywords:** Preterm infant, Bronchopulmonary dysplasia, Hyperoxia, Lung, Mesenchymal stem cells, FGF-10, HGF, IGF-1, PDGF-AA, Phenotype, Cell death

## Abstract

**Background:**

Phenotype disruption of lung resident mesenchymal stem cells (MSC) is a key event in the pathogenesis of bronchopulmonary dysplasia (BPD). Hyperoxia (HOX) constitutes one major harmful factor resulting in growth arrest and changes of functional properties including downregulation of PDGFRα. FGF-10, HGF, IGF-1 and PDGF-AA constitute promising cytokines to preserve lung growth. We studied their preventive application before HOX exposure of MSC cultures.

**Results:**

As described before, HOX inhibited spontaneous proliferation of MSC and induced cell death. Growth inhibition was larger at HOX80% than at HOX40%. The one-time preventive application of FGF-10, HGF, IGF-1 or PDGF-AA in therapeutic dosage or the repetitive application during HOX (40% or 80%) did not attenuate the growth inhibition and cell death induction by HOX even during the milder HOX40% exposure. Furthermore, the phenotype disruption with downregulation of PDGFRα as critical hallmark event remained unchanged.

**Conclusion:**

Our data indicate that the deleterious effects of HOX to lung resident MSC are that pronounced that single lung growth promoting cytokines cannot attenuate their phenotype disruption, growth inhibition and cell death by HOX. The results demand focus on MSC functionality when the therapeutic potential of lung growth promoting factors and other therapeutics intended to prevent BPD is studied.

**Supplementary Information:**

The online version contains supplementary material available at 10.1186/s40348-026-00241-4.

## Introduction

The histopathology of bronchopulmonary dysplasia (BPD) in the preterm infant is characterized by disruption of further lung development after birth. Hyperoxia (HOX), mechanical ventilation (MV) and pre- and postnatal infections constitute major contributors to the disease [1, 2]. Best studied for HOX in the preclinical models, oxygen toxicity induces a pulmonary inflammatory response and direct toxic effects that inhibit further alveolar and lung vascular development and affect the composition of the lung mesenchyme with rarefication and dysfunctionality of lung resident mesenchymal stem cells (MSC) that are diffusely scattered in the tissue and no longer positioned at the tip of the secondary septa [1]. Preclinical studies in newborn mice exposed to hyperoxia univocally prevailed a rarefication of platelet derived growth factor α (PDGFRα) positive MSC in the lungs and the pathology was more pronounced with higher concentrations of HOX [1, 3, 4]. Comparable rarefication was described in preterm human lung samples [5, 6]. Studies in PDGFRα knockout mice prevailed neonatal lethality due to disruption of lung development and newborn mice heterozygous for PDGFRα that are postnatally viable had more pronounced lung pathology than the wildtype animals following MV with oxygen rich gas. Molecular studies detailed the reduced lung resident MSC functionality in the context of PDGFRα signaling [5, 7]. Overall, the experimental evidence in rodents solidified the key role of MSC for alveolar epithelial and for vascular development [5, 7–10].

Studies in the preterm infant requiring MV after birth prevailed univocally that the phenotype disruption of lung resident MSC following exposure to MV with oxygen rich gas is the hallmark event associated with the development of moderate or severe BPD [5, 11, 12]. This pathological process is accompanied by distinct inflammatory signatures, including altered monocyte subpopulations that can serve as early predictors of BPD development (1). These severity stages of BPD pose the infants at high risk for life-long relevant restrictions in lung function [13]. MSC of preterm infants with BPD had higher phospho-GSK-3β/β-catenin and increased TGF-β signaling suggesting premature myofibroblast differentiation that counteracts NFκB signaling as lung growth and survival promoting pathway [1, 14]. But as for TGF-β, overshooting activation of NFκB by pro-inflammatory cytokines like IL1β or TNFα had detrimental effects on MSC phenotype which underpins the balance of signaling pathways as critical prerequisite for proper MSC functionality and lung development [1, 3, 5, 11, 14]. Recent evidence supports the central role of TNFα in this context, as distinct monocyte signatures with elevated TNFα signaling have been identified as early predictors of BPD development (1). Lastly, downregulation of PDGFRα was detected in MSC of preterm infants that were exposed to prolonged MV with oxygen rich gas that confirmed the relevance of these findings from the rodent models for the preterm infant [5]. We recently detailed the consequences of MSC exposure to HOX and cyclic mechanical stretch as correlate of MV in an in vitro scenario [15]. The exposures resulted in growth inhibition, cellular senescence, increased cell death induction and phenotype disruption that was mediated by p21 and went along with downregulation of PDGFRα [16]. Of clinical importance, the strength and duration of the exposures is critical for the irreversibility of the phenotype alteration [11, 16].

The approach of therapeutic application of recombinant lung growth promoting cytokines has attracted particular attention during the recent years based on the pathomechanistic understanding of a growth factor and cytokine disbalance with downregulation of lung growth promoting factors [1, 2, 4]. Within the panel of cytokines, insulin-like growth factor 1 (IGF-1) is the only one so far tested within a phase II trial based on the knowledge that preterm infants have reduced IGF-1 levels during their first weeks of life that is associated with the development of BPD [17]. The study was intended to prevent retinopathy of prematurity but prevailed efficient to reduce the risk and severity of BPD in line with the experimental evidence in the newborn hyperoxia rodent model [18, 19]. Fibroblast-like growth factor 10 (FGF-10) is another cytokine critical for lung development, particularly in the saccular stage and regulates cell proliferation and differentiation in the mesenchyme and epithelium [20, 20–22]. Based on the finding, that FGF-10 levels are reduced in the lungs of preterm infants FGF-10 deletion reporter mice strains and mice overexpressing FGF-10 were exposed to hyperoxia and prevailed that FGF-10 improves alveolar and vascular development [22–25]. For hepatocyte growth factor (HGF), reduced levels after birth were detected in preterm infants developing BPD [21, 26]. From a pathomechanistic perspective, HGF is required for vascular endothelial cell proliferation and alveolarization, is directly released by resident MSC and has the capacity to promote alveolar regeneration after lung injury and to reduce lung fibrosis by TGFβ1 [27–32]. Lastly, the PDGFRα signalling pathway draw particular attention based on the before described pathomechanistic understanding of its critical role for MSC functionality [3, 5–7, 14, 16]. When newborn PDGFRα haploinsufficient mice exposed to MV with oxygen rich gas were treated with recombinant PDGF-AA, the severity of lung injury was reduced and lung resident MSC morphometry and functionality better preserved when the latter was studied in in vitro assays [6].

Here, we expanded our pathomechanistic studies on hyperoxic (HOX) injury of lung resident MSC from preterm infants [5, 6, 16] to the therapeutic application of lung growth promoting factors IGF-1, FGF-10, HGF and PDGF-AA.

## Materials and methods

### Cell culture of lung resident MSC from preterm infants < 30 weeks´ gestation

MSC cultures were established from tracheal aspirates of preterm infants < 30 weeks of gestation as described before [11, 16]. Shortly, tracheal aspirates were centrifuged at 2000 g for 5 min and pellets were resuspended and taken into cell culture on 96 well plates in MesenCult medium (Stem Cell Technologies, Vancouver, Canada) supplemented with 20% fetal calf serum (FCS, PAN Biotech Aidenbach, Germany or Invitrogen, Carlsbad, CA), 10 mM HEPES buffer solution and 50U/ml penicillin, 50 µg/ml streptomycin and 50 µg/ml gentamycin (Invitrogen). MSC were expanded into cell culture flasks in DMEM medium (41,965,092, Thermo Fisher, Waltham, US) now supplemented with 10% FCS (10,270,106, Thermo Fisher, Waltham, US) and split when confluency was > 75% by visual assessment. Purity of MSC cultures was > 95% as determined by flow cytometry with positivity for CD73 and CD90 and negativity for CD45 as done before [11, 16]. All experiments with the human preterm MSC were performed according to the principles of the Helsinki declaration and with site approval by the ethics committee of the Justus-Liebig-University Gießen (Az. 135/12). The study was registered at Deutsches Register Klinische Studien (DRKS00004600). Written informed parental consent was obtained from the parents of all participating infants.

### Experimental approach

For experimental readouts, MSC were plated on 6 well plates the day before the start of the experiment with a cell count of 100.000 cells per well for FACS analysis and 200.000 cells per well for protein analysis. 24 h prior to start of exposure to room air (normoxia, NOX), HOX40% or HOX80% for 72 h, MSC were stimulated with either recombinant IGF-1 (100 ng/ml), FGF-10 (10 ng/ml), HGF (10 ng/ml) or PDGF-AA (10 ng/ml) from Pepro Tech, Hamburg, Germany). The cytokine dosages used were selected according to the published literature on the topic [33–37]. Recombinant cytokines were either applied once or application was repeated every 24 h at the equivalent concentration. Constant application of HOX was monitored within the self-constructed chambers using a GOX 100 oxygen sensor (Greisinger electronic GmbH, Regenstauf, Germany) and a maximum variation of ± 5% was permitted.

### Determination of cell counts and cell death induction

Flow cytometry based analyses were executed on a BD FACS CANTO II (Becton Dickinson, Franklin Lakes, US) using Diva software (version 6.1.3, Becton Dickinson, Franklin Lakes, US) for data acquisition. Analyses of data were done with FlowJo software (version 10.7.1, Becton Dickinson, Franklin Lakes, US). Quantification of MSC was done after addition of 5000 cell counting beads (ACFP-100–3, Spherotec, Lake Forest, US) per sample. Cell death induction was determined with an Annexin V (640,947, PE conjugated, Biolegend, San Diego, US) and SYTOX Blue (S34857, Thermo Fisher, Waltham, US) double staining. Staining was performed on ice after resuspension with PBS in Annexin V Binding Buffer (422,201, Biolegend, San Diego, US) for 30 min. Directly before the analyses, SYTOX blue dye was added. Quantification of cell death was calculated as the quotient of [dead cells/frequence of all single cells].

### Western blot analysis

Protein analysis was done after lysis of MSC with RIPA lysis buffer (24,948, Santa Cruz Biotechnology, Dallas, US). Pierce BCA Protein Assay Kit (23,225, Thermo Fisher, Waltham, US) was used for determination of protein concentration and measured by Nanodrop technology. Denaturation was done with Laemmli buffer containing 2-mercaptoethanol for 5 min at 95 °C and protein extract was loaded on a 10% SDS-gels followed by protein transfer onto nitrocellulose membranes (0.2 µm pore size). Unspecific protein binding was blocked with 5% dry milk in TBS + 2.5% Tween (TBS-T) at room temperature for 1 h. Primary antibodies were incubated overnight at 4 °C in the blocking buffer on a roller in closed tubes. After three washes with TBS-T, membranes were incubated with the respective HRP-labelled secondary antibodies at room temperature for 1 h followed by another three washes with TBS-T buffer. For immunoblot analyses, the following primary antibodies were used: αSMA (58,669, Santa Cruz, Dallas, US, mouse-monoclonal, 1:5.000), PDGFRα (3174S, Cell Signaling, Danvers, US, rabbit monoclonal, 1:1.000) and GAPDH as housekeeper (MAB37437, Merck, Darmstadt, DE, mouse monoclonal, 1:5.000). HRP-conjugated secondary antibodies were anti-rabbit (7074S, Cell Signaling, Danvers, US, 1:1.000) and anti-mouse (sc-516102, Santa Cruz, Dallas, US, 1:1.000) IgG. Chemiluminescence was induced by the SuperSignal West Femto kit (A38554, Thermo Fisher, Waltham, US) and signal detection done using the ChemiDoc XRS + Gel Imaging System (Biorad, Hercules, US). Signal quantification was performed using ImageLab (version 6.0, Biorad, Hercules, US) and calculation of lane normalization factor as described previously [16]. All gels were normalized to control-intervention group. The order of samples in some blots was rearranged for the clearness of presentation without any further manipulation (indicated by separated boxes). The bar graphs show the densitometry results expressed as the ratio of adjusted total band volume normalized to GAPDH and control. Original Western blot data can be found in the appendix (supplemental Figure S1).

### Calculation of cell proliferation and statistical analysis

The cell expansion index (CEI) was calculated as the quotient of [living cell count at the end of the experiment/living cell count at the start of the experiment] relying on the flow cytometry measurements. Thereof, the change in CEI by the exposures of HOX and/or lung growth promoting factor was calculated compared to the spontaneous proliferation as (CEI exposure/CEI control) – 1. Thus, in our experimental setting the CEI reflects the increase in the number of living cells from the start to the end of the observation as detailed previously [16].

For all experimental settings, data are given as median and interquartiles. Two-way ANOVA with Bonferroni multiple comparisons test was used to test for statistically significant differences. BioRender (Toronto, CAN) and Sigma Plot (version 12.3, Systat Software, San Jose, CA) was used for calculation and data presentation. Differences were considered significant at *p*-values < 0.05.

## Results

Based on our recent results that HOX induces severe MSC phenotype changes, growth arrest and cell death induction particularly when applied at HOX80%, we now studied the therapeutic potential of preventive application of the lung growth promoting cytokines IGF-1, FGF-10, HGF and PDGF-AA. We preferred the preventive approach although not feasible in the clinics where HOX exposure starts immediately after birth in the delivery room not to miss any beneficial effect of the intervention that might be missed with the retarded therapeutic intervention after start of HOX. We included the strong exposure to HOX80% and the much milder to HOX40% where the extent of MSC phenotype changes is much less pronounced in vitro and in vivo and preterm infants have a better pulmonary outcome [3, 16, 38]. Baseline characteristics of preterm infants whose MSC cultures were included into the studies are summarized in Table 1. As in our previous publication, preterm infants had a very low gestational age (mean 24 + 3 weeks GA, IQR 23 + 2–27 + 5 weeks GA) and birth weight (mean 786 g, IQR 551–1147 g) with nearly equal gender distribution and variable severity stages of BPD (Table 1 and individual patient data in supplemental Table S1).

**Table 1:** Demographics of the study collective (*n* = 4)

variables	mean (IQR) or n (%)
GA, weeks	24 + 3 (23 + 2—27 + 5)
BW, g	786 (551—1147)
Male	2 (50.0)
Multiples	1 (25.0)
SGA	0 (0.0)
Chorioamnionitis	1 (25.0)
ANCS	4 (100.0)
IMV, days	15.75 (5–26.5)
IVH (any grade)	1 (25.0)
ROP (any grade)	3 (75.0)
FIP	2 (50.0)
NEC	0 (0.0)
BPD (any severity)	3 (75.0)
Mild	0 (0.0)
Moderate	2 (50.0)
Severe	1 (25.0)
Death (before discharge)	0 (0.0)

Perinatal and outcome characteristics of the n = 4 preterm infants whose resident lung MSC cultures were used in the study. Data are presented as mean (IQR; interquartile range) or n (%)

*GA* Gestational age, *BW* Birthweight, *SGA* Small for gestational age, *ANCS* Antenatal corticosteroids, *IMV* Invasive mechanical ventilation, *IVH* Intraventricular hemorrhage, *ROP* Retinopathy of prematurity, *FIP* Focal intestinal perforation, *NEC* Necrotizing enterocolitis, *BPD* Bronchopulmonary dysplasia

### Preventive application of IFG1, FGF-10, HGF and PDGF-AA and growth inhibition of MSC by HOX

**Fig. 1:**
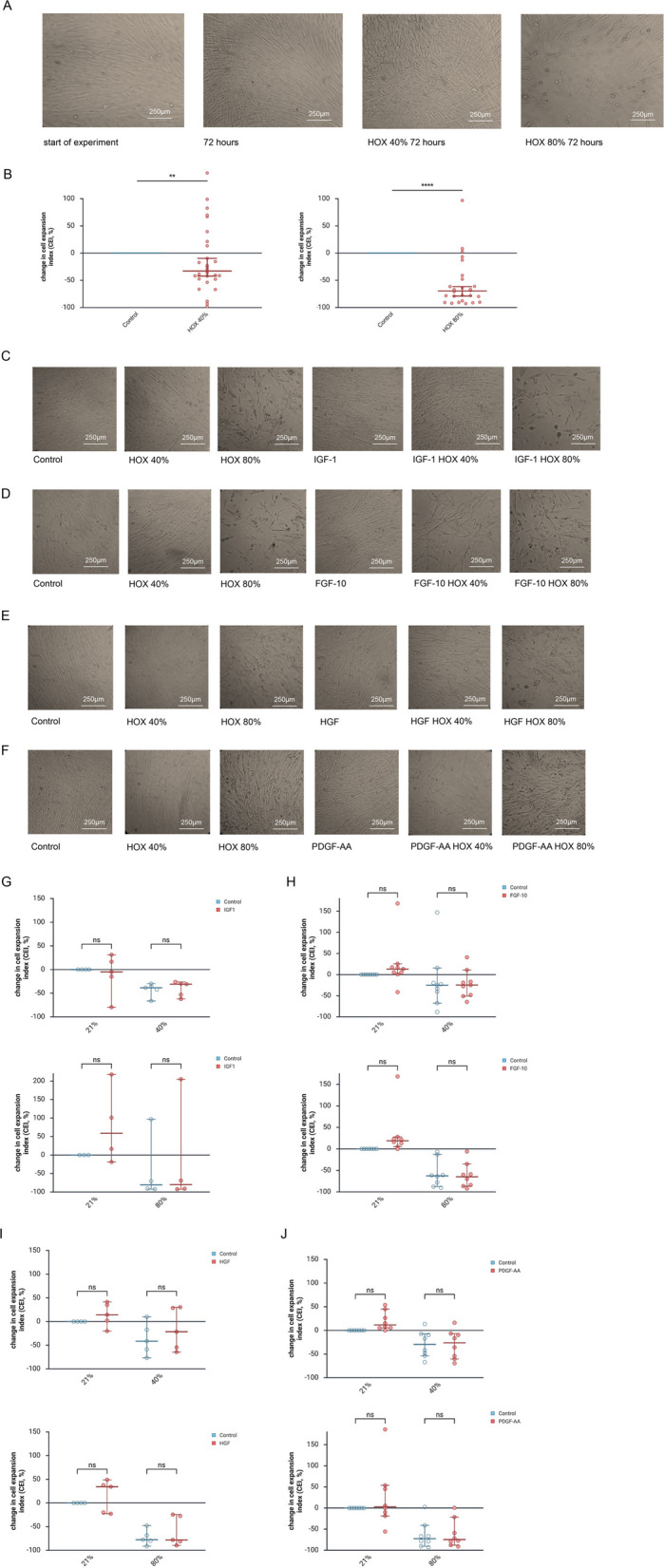
Preventive application of lung growth promoting cytokines before hyperoxia (HOX) exposure of lung resident mesenchymal stem cells (MSC) of preterm infants and cell growth. **A** Bright-field microscopy pictures at start of the experiment and after 72 h of exposure to hyperoxia (HOX40% or HOX80%). **B** Change in cell expansion index (CEI) by hyperoxia (HOX40% and HOX80%) compared to the proliferation of controls measured by flow cytometry of living cells. HOX exposure led to a dose-dependent inhibition of MSC proliferation. **C-F** MSC were preincubated with IGF-1 (**C**, 100 ng/ml), FGF-10 (**D**, 10 ng/ml), HGF (**E**, 10 ng/ml) or PDGF-AA (**F**, 10 ng/ml) 24 h before exposure to HOX40% or HOX80% for another 72 h. Representative bright-field microscopy pictures are displayed. **G-J** Independent wells were stimulated in parallel as in (**C**-**F**). Change in CEI is calculated and presented as in (**B**). Preventive growth factor application does not reduce the growth inhibition of MSC by the two HOX conditions. **A** and **C-F**. One representative out of *n* = 3 independent experiments is displayed. **B** Analysis of *n* = 28 (HOX40%) and *n* = 26 (HOX80%) independent experiments from *n* = 4 different MSC cultures, **G** Analysis of *n* = 5 independent experiments from *n* = 2 different MSC cultures, **H** Analysis of *n* = 9 (HOX40%) and *n* = 8 (HOX80%) independent experiments from *n* = 4 different MSC cultures, **I** Analysis of *n* = 5 independent experiments from *n* = 2 different MSC cultures, **J** Analysis of *n* = 9 (HOX40%) and *n* = 8 (HOX80%) independent experiments from *n* = 4 different MSC cultures. Data are expressed as median with 95% confidence interval. Two-way ANOVA with Bonferroni multiple comparisons test was used to calculate statistically significant differences. **p < 0.01, ****p < 0.0001

Early passage MSC cultures exposed to HOX 40% or 80% displayed the characteristic inhibition of proliferation that became already evident during light microscopy and was objectively measured using flow cytometry and cell counts on living cells. As described before, growth inhibition was more pronounced for HOX80% than for HOX40% (Fig. 1A and B) [16]. One-time preventive application of none of the four growth factors prevailed a visual or measurable benefit on the MSC count of living cells (Fig. 1C-J). The result remained unchanged when the growth factors were applied repetitively every 24 h during the intervention (supplemental Figure S2A-D).

### Preventive treatment with IFG1, FGF-10, HGF and PDGF-AA and cell death induction of MSC by HOX

**Fig. 2:**
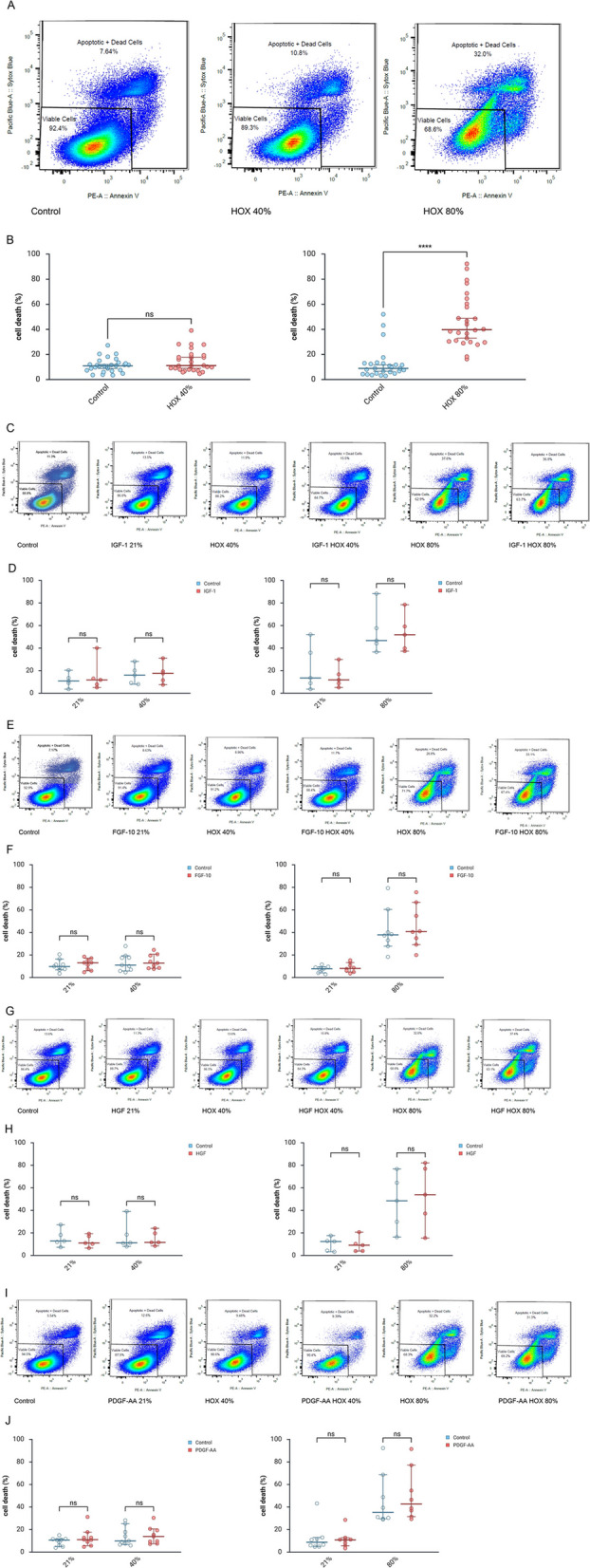
Preventive application of lung growth promoting cytokines before hyperoxia (HOX) exposure of lung resident mesenchymal stem cells (MSC) of preterm infants and cell death induction. **A** Flow cytometry data of cell death induction in lung resident MSC following 72 h of exposure to hyperoxia (HOX40% or HOX80%). **B** Absolute cell death induction measured by flow cytometry applying Annexin V and SYTOX blue double staining. HOX80% but not HOX40% increased the cell death induction of MSC. **C**, **E**, **G**, **I** MSC were preincubated with IGF-1 (**C**, 100 ng/ml), FGF10 (**D**, 10 ng/ml), HGF (**E**, 10 ng/ml) or PDGF-AA (**F**, 10 ng/ml) 24 h before exposure to HOX40% or HOX80% for another 72 h. Representative flow cytometry analyses of cell death induction are displayed. **D**, **F**, **H**, **J** Comparison of cell death induction by HOX in the absence or presence of the growth factors as in (**C**, **E**, **G**, **I**). Preventive growth factor application does not reduce the cell death of MSC by HOX80%. **A** and **(C**, **E**, **G**, **I)** One representative out of *n* = 3 independent experiments is displayed. **B** Analysis of *n* = 28 (HOX40%) and *n* = 26 (HOX80%) independent experiments from *n* = 4 different MSC cultures, **D** Analysis of *n* = 5 independent experiments from *n* = 2 different MSC cultures, **F** Analysis of *n* = 9 (HOX40%) and *n* = 8 (HOX80%) independent experiments from *n* = 4 different MSC cultures, **H** Analysis of *n* = 5 independent experiments from *n* = 2 different MSC cultures, **J** Analysis of *n* = 9 (HOX40%) and *n* = 8 (HOX80%) independent experiments from *n* = 4 different MSC cultures. Data are expressed as median with 95% confidence interval. Two-way ANOVA with Bonferroni multiple comparisons test was used to calculate statistically significant differences. ****p < 0.0001

As in our previous study, cell death induction by HOX40% was only marginal and < 10% in the rate but increased to 40% when HOX80% was applied for 72 h (Fig. 2A and B). In line with the studies on growth inhibition, the preventive application of none of the four cytokines did significantly reduce the cell death induction by HOX (Fig. 2C-J). The results remained unchanged when the growth factors were added daily (supplemental Figure S3A-D).

### Preventive IFG1, FGF-10, HGF and PDGF-AA and changes in MSC marker protein expression by HOX exposure

**Fig. 3:**
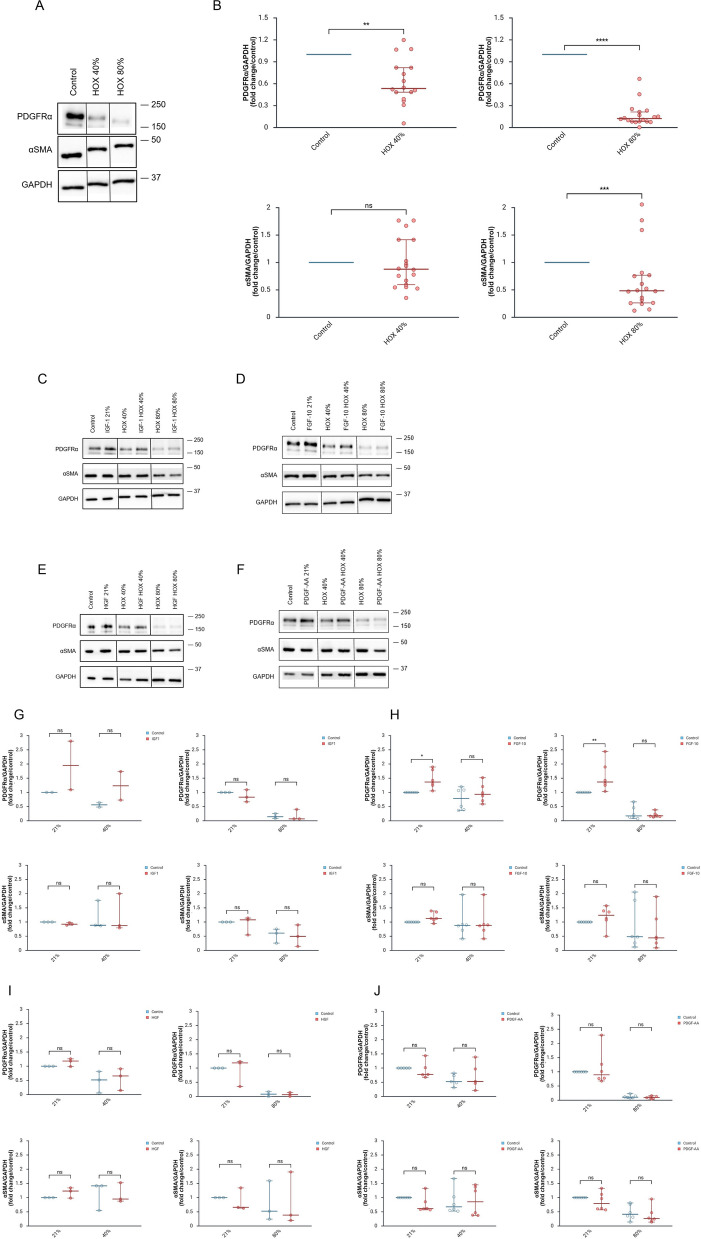
Preventive application of lung growth promoting cytokines and hyperoxia mediated changes in the expression of lung resident MSC marker proteins. **A** Western blot analysis for PDGFRα, αSMA and GAPDH as loading control of MSC exposed to HOX40% and HOX80%. **B** Change in marker protein expression comparing normoxia, HOX40% and HOX80% exposures. HOX reduced the expression of PDGFRα for both HOX exposures (40% and 80%), while αSMA protein was downregulated with HOX80%. **C-F** MSC preincubated with IGF-1 (**C**, 100 ng/ml), FGF10 (**D**, 10 ng/ml), HGF (**E**, 10 ng/ml) or PDGF-AA (**F**, 10 ng/ml) 24 h before exposure to HOX40% or HOX80% for another 72 h were analyzed for PDGFRα, αSMA and GAPDH expression. **G-J** Comparison of PDGFRα and αSMA normalized to GAPDH expression following HOX exposures in the absence or presence of the growth factors as in (**C**, **E**, **G**, **I**). Preventive growth factor application does not preserve the reduction of PDGFRα and αSMA levels in MSC by HOX. **A** and (**C-F**) One representative out of *n* = 3 independent experiments is displayed. **B** Analysis of *n* = 4 different MSC cultures (PDGFRα HOX40% *n* = 16, HOX 80% *n* = 18; αSMA *n* = 18 independent experiments), **G** Analysis of *n* = 2 different MSC cultures (PDGFRα HOX40% *n* = 2, HOX 80% *n* = 3; αSMA *n* = 3 independent experiments), **H** Analysis of *n* = 4 different MSC cultures (*n* = 6 independent experiments), **I** Analysis of *n* = 2 different MSC cultures (*n* = 3 independent experiments), **J** Analysis of *n* = 4 different MSC cultures (PDGFRα HOX40% *n* = 5, HOX 80% *n* = 6; αSMA *n* = 6 independent experiments). The order of samples in some blots was rearranged for the clearness of presentation without any further manipulation (indicated by separated boxes). The bar graphs show the densitometry results expressed as the ratio of adjusted total band volume normalized to GAPDH and control. Data are expressed as median with 95% confidence interval. Two-way ANOVA with Bonferroni multiple comparisons test was used to calculate statistically significant differences. **p < 0.01, ***p < 0.001, ****p < 0.0001

During our recent studies we had identified PDGFRα and αSMA as those MSC marker proteins with the strongest regulation following hyperoxic or inflammatory exposures while CD cell surface marker proteins were largely unchanged [11, 16]. As described before, baseline expression of PDGFRα and αSMA differed largely between the different MSC cultures [11]. And HOX80% induced more pronounced changes on the protein level than HOX40% as could be expected from the previous study on the topic but in this experimental series there was a significant reduction in αSMA expression (Fig. 3A + B) [16]. The preventive application of none of the four lung growth promoting cytokines IGF-1, FGF-10, HGF and PDGFA induced any change in the HOX mediated downregulation of PDGFRα and αSMA on the protein level (Fig. 3C-J). Again, the results remained unchanged when growth factors were added daily into the cell cultures (supplemental Figures S4A-D).

### Combinatorial pretreatment with FGF-10 and PDGF-AA

**Fig. 4:**
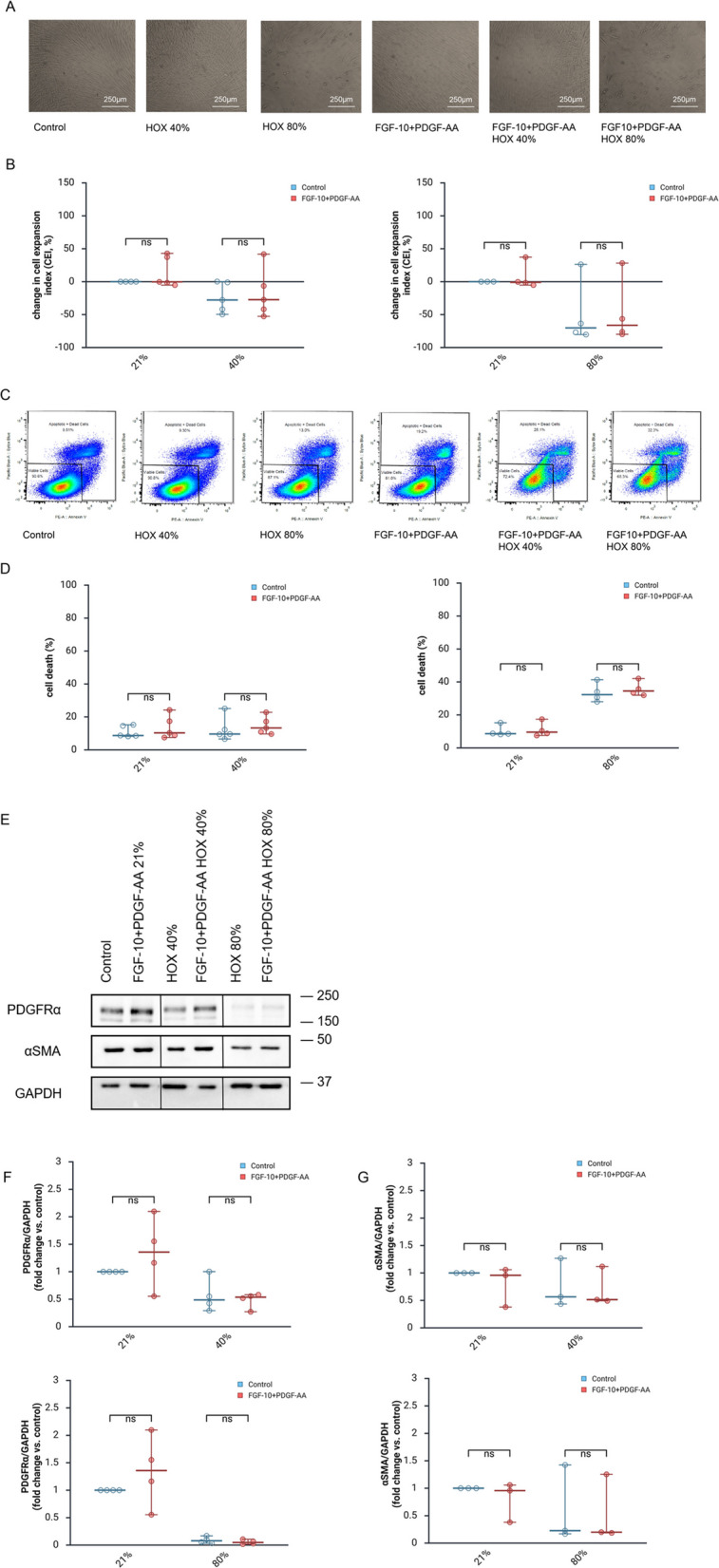
Combinatorial pretreatment with FGF-10 and PDGF-AA. **A** MSC were preincubated with combination of FGF-10 (10 ng/ml) and PDGF-AA (10 ng/ml) 24 h before exposure to HOX40% or HOX80% for another 72 h. Representative bright-field microscopy pictures are displayed. **B** Change in cell expansion index (CEI) by hyperoxia (HOX40% and HOX80%) compared to the proliferation of controls measured by flow cytometry of living cells. HOX exposure led to a moderate dose-dependent inhibition of MSC proliferation. Preventive combinatorial growth factor application does not reduce the growth inhibition of MSC by the two HOX conditions. **C** Flow cytometry data of cell death induction in lung resident MSC preincubated with combination of FGF-10 (10 ng/ml) and PDGF-AA (10 ng/ml) 24 h before exposure following 72 h of exposure to hyperoxia (HOX40% or HOX80%). Absolute cell death induction measured by flow cytometry applying Annexin V and SYTOX blue double staining. Representative flow cytometry analyses of cell death induction are displayed (**D**). Preventive combinatorial growth factor application does not reduce the cell death of MSC by HOX40% or HOX80%. **E** Western blot analysis for PDGFRα, αSMA and GAPDH as loading control of MSC after preincubation with combination of FGF-10 (10 ng/ml) and PDGF-AA (10 ng/ml) 24 h before exposure to HOX40% or HOX80% for another 72 h. **F-G** Comparison of PDGFRα and αSMA normalized to GAPDH expression following HOX exposures in the absence or presence of the combined growth factors. Preventive combinatorial growth factor application does not preserve the reduction of PDGFRα and αSMA levels in MSC by HOX. **A**,**C**,**E** One representative out of *n* = 3 independent experiments is displayed. **B**,**D** Analysis of *n* = 5 (HOX40%) and *n* = 4 (HOX80%) independent experiments from *n* = 3 different MSC cultures. **F** Analysis of *n* = 4 independent experiments from *n* = 3 different MSC cultures. **G** Analysis of *n* = 3 independent experiments from *n* = 3 different MSC cultures. Data are expressed as median with 95% confidence interval. Two-way ANOVA with Bonferroni multiple comparisons test was used to calculate statistically significant differences

Lastly, we tested the combinatorial pretreatment with FGF-10 plus PDGF-AA. As for the single cytokine application, no alteration in growth inhibition, cell death induction and MSC marker protein expression induced by HOX40% and HOX80% were detected for the one-time preventive (Fig. 4A-G) and the repetitive (supplemental Figure S5A-C) application.

## Discussion

HOX constitutes the most detrimental injury to the immature lung within the postnatal exposures and NICU environment. Recommendations of clinical treatment guidelines around the world result in HOX exposure to all preterm infants during their stay in the NICU to safeguard their survival while the intrauterine milieu is hypoxic and thereby stipulates lung development in the late canalicular and saccular stage [1, 4, 39]. For these reasons, researchers endeavor a targeted intervention that preserves physiologic lung development *ex utero*. Based on the promising results from the preclinical studies in the rodent models, we selected 4 candidate cytokines that seemed particularly attractive based on the experimental evidence or have already entered the stage of clinical studies as holds true for IGF-1 [19]. Despite choosing the most beneficial preventive approach of application before the start of the HOX exposure we did not detect any benefit for the growth promoting capacities, survival and marker protein expression of lung resident MSC obtained from preterm infants in our in vitro setting that were hardly impacted by direct HOX injury. The results were comparable for MSC cultures from male and female sex and from infants with a better or worse pulmonary outcome (data not shown). Thereby, the data indicate their general applicability to the population of preterm infants. Disappointing to conclude that not even a trend towards a benefit became available in our studies for any of the four highly promising candidates IFG1, FGF-10, HGF and PDGF-AA based on the available data and the proposed mechanisms of action.

By testing the exposures to HOX40% and HOX80% we simulated both the situation of preterm infants with milder and severe disturbance of gas exchange and the necessary exposure of their lungs to lower or higher concentrations of oxygen [38]. The studies on lung resident MSC cultures enabled studies of the direct effects of HOX on MSC in the absence of amplification by the pulmonary inflammatory immune response which is a relevant fraction of HOX toxicity [1–4]. Thereby, we were able to specify the direct HOX effects on MSC. But our therapeutic intervention was not able to respect the other cell types of the lung and the recruitment of immune cells following HOX. This is why we might have missed beneficial effects of the four cytokines on other lung cell types as have been described for the epithelium and vascular endothelium or indirectly via modulating the inflammatory response [22, 24]. This might explain the discrepancy between the beneficial effects in the rodent studies and our human cell culture model. But on the other hand, large differences exist in therapeutic efficacy between rodent and human outcome studies for a magnitude of interventions and our data might add further candidates to this list [40].

We must acknowledge limitations to our study. The study outline was designed to test predefined outcomes within the simplified experimental setting of isolated HOX exposure and did not apply unbiased approaches and could not respect the complex in vivo scenario of the various exposures including mechanical ventilation. Thereby, we might have missed positive effects beyond the classical readouts in our lab. Furthermore, due to the study approach we were not able to run the experiments on a large magnitude of MSC cultures but only on *n* = 4 different samples and all with moderate/severe BPD and might have missed minor changes. But as there were uniform outcomes during all previous studies on MSC cultures in vitro in our and other labs and relevant results became not only evident but significant with three to eight replications on independent MSC cultures this situation is unlikely for outcomes that apply to the general population of preterm infants [6, 11, 12, 16]. One might furthermore argue that the MSC were obtained from the lungs of preterm infants that had already been exposed to pro-inflammatory stimuli. Indeed this holds true for HOX and MV and partly for infectious stimuli but it must be noted that all resident MSC get postnatally exposed to one or the other form of inflammatory stimulus and thereby a therapy will not be effective to the majority of preterm infants that is only effective when applied before start of the deleterious exposures. And we demonstrated before, that the induced MSC phenotype changes are in principle reversible [11, 16]. Lastly, we did not test cytokine levels during the experimental procedures in the cell culture medium and thereby cannot exclude a rapid decline during the HOX exposure. But we i) applied repetitive application every 24 h and ii) checked known readouts for cytokine functionality including the upregulation of PDGFRα by FGF-10 and PDGF-AA [6, 41]. Therefore, we retrieved positive expected readouts that were abolished by HOX in a situation of isolated MSC cell exposure where for example protease action following the release by neutrophils and other cells of the inflammatory response in the immature lung is excluded [1].

### Concluding remarks

In summary, our studies on the therapeutic potential of single lung growth promoting cytokines in the context of HOX injury as the strongest stimulus and toxic exposure to the immature lung did not prevail any therapeutic benefit for this specific cell population [3, 4, 39, 42]. Our results although disappointing at first sight should not prohibit further studies on the therapeutic potential of further lung growth promoting factors like VEGFA, EGF or CTGF that are deficient in the lungs of preterm infants. Rather, the magnitude of changes in these cytokines following the injurious exposures indicates that it is not sufficient to substitute one single cytokine [1]. Future research directions shall focus on the combination of several of the most promising candidates and shall include the focus on the other compartments of the lung that was out of the focus of our current study. Allogenic MSC application might constitute a promising alternative that unite the secretion of a plenty of lung growth promoting factors with the release of anti-inflammatory cytokines that have beneficial effects from two different sides [2, 43]. And the repetitive application although not of advantage in our setting is particularly promising as it overcomes the inactivation of the beneficial factors and the loss of functional properties of exogenous MSC in the inflamed lung [43, 44]. But the local microenvironment and exposure to MV and HOX might limit their functionality as published recently [44, 45]. It will be imperative for any future intervention study particularly when a targeted intervention to preserve or rescue lung growth is intended to respect and focus on the MSC functionality across ages as the implications of our study outreach the neonatal period and the data should encourage lung researchers faced with HOX injury or the need for tissue regeneration to consider our findings in the design of their research.

## Supplementary Information

Supplementary Material 1.

## Data Availability

All data generated or analyzed during this study are included in this published article and its supplementary information files. The supplementary material is available at the online public repository *figshare* via: 10.6084/m9.figshare.31276966).

## Abbreviations

BPD: Bronchopulmonary dysplasia
CEI: Cell expansion index
FCS: Fetal calf serum
FGF-10: Fibroblast-like growth factor 10
HGF: Hepatocyte growth factor
HOX: Hyperoxia
IGF-1: Insulin-like growth factor 1
MSC: Mesenchymal stem cells
MV: Mechanical ventilation
NOX: Normoxia
