# Supplementary material for: Preventive application of lung growth factors and lack of attenuation of phenotype disruption of lung resident MSC from preterm infants by hyperoxia
Source: Mol Cell Pediatr. 2026 May 22;13:28. doi: 10.1186/s40348-026-00241-4 (PMC13197496; doi:10.1186/s40348-026-00241-4)
Supplement: Supplementary file 1 — Supplementary Material 1. [file 40348_2026_241_MOESM1_ESM.pdf]

SUPPLEMENTAL MATERIAL

Preventive application of lung growth factors and lack of attenuation of phenotype disruption of lung resident MSC from preterm infants by hyperoxia

Lena Holzfurtner[1+], Judith Behnke[1+], Pauline Korte[1], Maurizio J Goetz[1], Jutta Petzinger[1], Anita C Windhorst[2], Tayyab Shahzad[1], Stefano Rivetti[3], Ying Dong[1,4], Saverio Bellusci[3,5], Harald Ehrhardt[1,4,6*]

[1] Department of General Pediatrics and Neonatology, Justus-Liebig-University Giessen and Universities of Giessen and Marburg Lung Center (UGMLC), Member of the German Center for Lung Research (DZL), 35392 Giessen, Germany.

[2] Institute of Medical Informatics (IMI), Justus-Liebig University Giessen, 35392 Giessen, Germany

[3] Justus-Liebig-University Giessen and Universities of Giessen and Marburg Lung Center (UGMLC), Excellence Cluster Cardio Pulmonary Institute (CPI), Member of the German Center for Lung Research (DZL), 35392 Giessen, Germany.

[4] Department of Neonatology, Charité – Universitätsmedizin Berlin, 13353 Berlin, Germany.

[5] Institute for Lung Health (ILH), 35392 Giessen, Germany

[6] German Centre for Child and Adolescent Health (DZKJ), partner site Berlin, Berlin, Germany

[+]shared first-authorship

Supplemental Table S1: Individual patient characteristics and number of passages

ID	GA (weeks)	BW (g)	gender	BPD severity stage	date of assimilation (postnatal day)	number of passages for experiment
1	23+4	750	male	severe	29	P3-P4
2	23+4	545	female	moderate	9, 10	P3-P6
3	23+5	570	male	moderate	9	P2
4	29+1	1280	female	no BPD	5	P2

GA – gestational age; BW – birth weight

Supplemental Figure Legends

S2: Daily preventive application of lung growth promoting cytokines while hyperoxia (HOX) exposure of lung resident mesenchymal stem cells (MSC) of preterm infants and cell growth.

Change in cell expansion index (CEI) by hyperoxia (HOX40% and HOX80%) compared to the proliferation of controls measured by flow cytometry of living cells. MSC were incubated with IGF-1 (A, 100ng/ml), FGF-10 (B, 10ng/ml), HGF (C, 10ng/ml) or PDGF-AA (D, 10ng/ml) every 24 hours during exposure to HOX40% or HOX80% for 72 hours. Repetitive growth factor application does not reduce the growth inhibition of MSC by the two HOX conditions.

(A) Analysis of n=5 independent experiments from n=2 different MSC cultures, **(B)** Analysis of n=9 (HOX40%) and n=8 (HOX80%) independent experiments from n=4 different MSC cultures, **(C)** Analysis of n=5 independent experiments from n=2 different MSC cultures, **(D)** Analysis of n=9 (HOX40%) and n=8 (HOX80%) independent experiments from n=4 different MSC cultures.

Data are expressed as median with 95% confidence interval. Two-way ANOVA with Bonferroni multiple comparisons test was used to calculate statistically significant differences.

S3: Daily preventive application of lung growth promoting cytokines before hyperoxia (HOX) exposure of lung resident mesenchymal stem cells (MSC) of preterm infants and cell death induction.

Comparison of cell death induction by HOX in the absence or presence of the growth factors via flow cytometry. MSC were incubated with IGF-1 (A, 100ng/ml), FGF10 (B, 10ng/ml), HGF (C, 10ng/ml), or PDGF-AA (D, 10ng/ml) every 24 hours during exposure to HOX40% or HOX80% for 72 hours. Daily preventive growth factor application does not reduce the cell death of MSC by HOX80%.

(A) Analysis of n=5 independent experiments from n=2 different MSC cultures, **(B)** Analysis of n=9 (HOX40%) and n=8 (HOX80%) independent experiments from n=4 different MSC cultures, **(C)** Analysis of n=5 independent experiments from n=2 different MSC cultures, **(D)** Analysis of n=9 (HOX40%) and n=8 (HOX80%) independent experiments from n=4 different MSC cultures,

Data are expressed as median with 95% confidence interval. Two-way ANOVA with Bonferroni multiple comparisons test was used to calculate statistically significant differences.

S4: Daily application of lung growth promoting cytokines and effect on hyperoxia mediated changes in the expression of lung resident MSC marker proteins.

MSC incubated with IGF1 (A, 100ng/ml), FGF10 (B, 10ng/ml), HGF (C, 10ng/ml) or PDGF-AA (D, 10ng/ml) every 24 hours during exposure to HOX40% or HOX80% for 72 hours were analyzed for PDGFRα, αSMA and GAPDH expression. Comparison of PDGFRα and αSMA normalized to GAPDH expression following HOX exposures in the absence or presence of the growth factors. Preventive growth factor application does not preserve the reduction of and αSMA levels in MSC by HOX.

(A) Analysis of n=2 different MSC cultures (PDGFRα HOX40% n=2, HOX 80% n=3; αSMA n=3 independent experiments), **(B)** Analysis of n=4 different MSC cultures (n=6 independent experiments), **(C)** Analysis of n=2 different MSC cultures (n=3 independent experiments), **(D)** Analysis of n=4 different MSC cultures (PDGFRα HOX40% n=5, HOX 80% n=6; αSMA n=6 independent experiments),

Data are expressed as median with 95% confidence interval. Two-way ANOVA with Bonferroni multiple comparisons test was used to calculate statistically significant differences.

S5: Daily Combinatorial pretreatment with FGF-10 and PDGF-AA

MSC were incubated with combination of FGF-10 (10ng/ml) and PDGF-AA (10ng/ml) every 24 hours during exposure to HOX40% or HOX80% for 72 hours.

(A) Change in cell expansion index (CEI) by hyperoxia (HOX40% and HOX80%) compared to the proliferation of controls measured by flow cytometry of living cells. Repetitive combinatorial growth factor application does not reduce the growth inhibition of MSC by the two HOX conditions. Analysis of n=5 (HOX 40%) and n=4 (HOX 80%) independent experiments from n=3 different MSC cultures.

(B) Comparison of cell death induction by HOX in the absence or presence of the growth factors via flow cytometry. Daily combinatorial preventive growth factor application does not reduce the cell death of MSC by HOX80%. Analysis of n=5 (HOX40%) and n=4 (HOX80%) independent experiments from n=3 different MSC cultures.

(C) Analysis of PDGFRα, αSMA and GAPDH expression. Comparison of PDGFRα and αSMA normalized to GAPDH expression following HOX exposures in the absence or presence of the growth factors. Preventive combinatorial growth factor application does not preserve the reduction of and αSMA levels in MSC by HOX.

Analysis of n=3 different MSC cultures (PDGFRα n=4; αSMA n=3 independent experiments).

Data are expressed as median with 95% confidence interval. Two-way ANOVA with Bonferroni multiple comparisons test was used to calculate statistically significant differences.

Supplemental Figure S1: Original Western blot data

Figure 3A

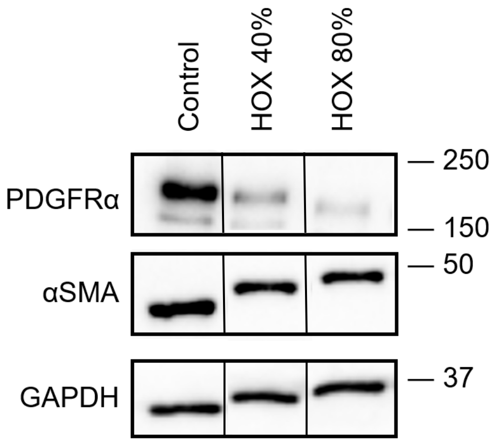

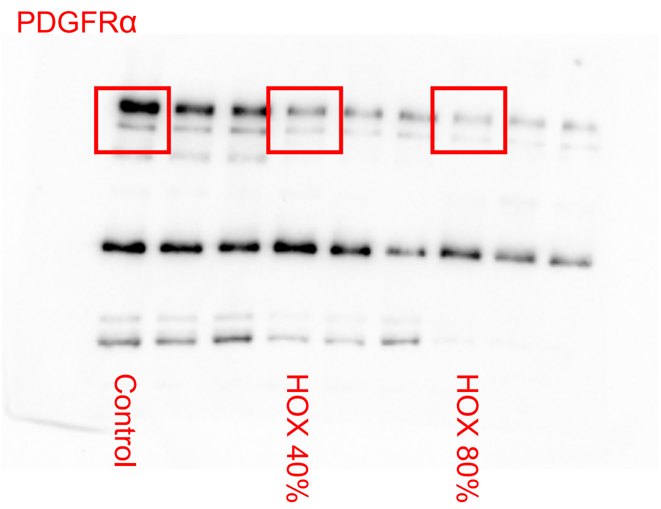

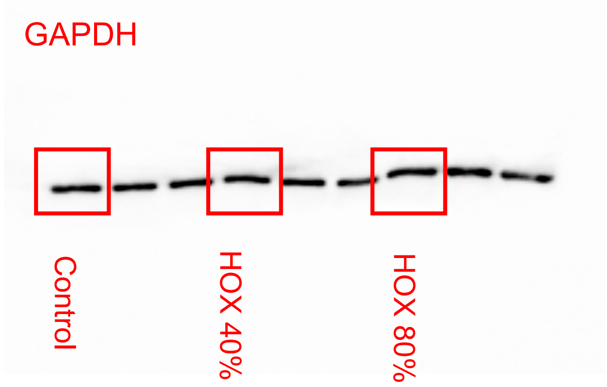

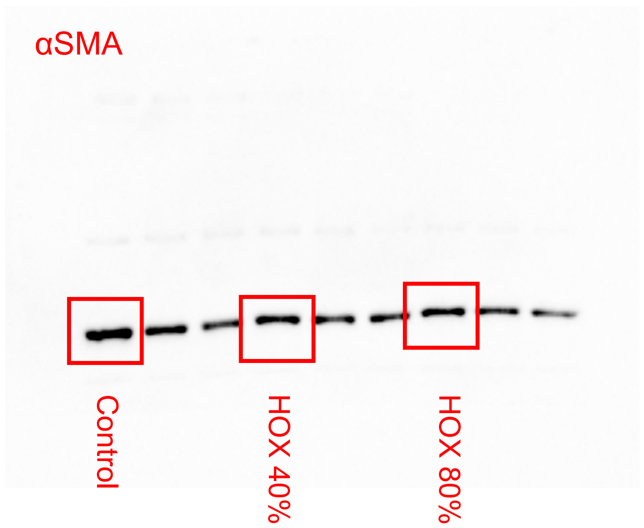

Figure 3C

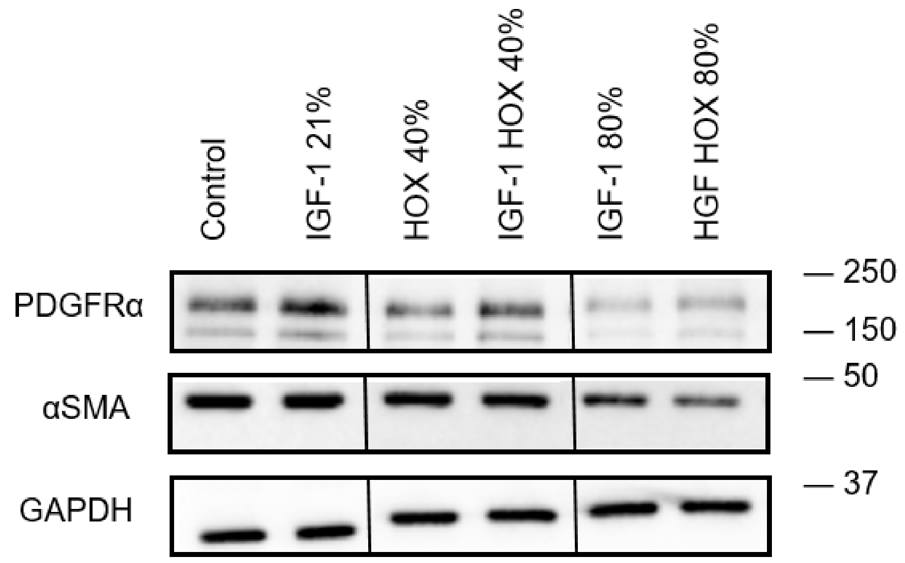
Control
IGF-1 21%
HOX 40%
IGF-1 HOX 40%
IGF-1 80%
HGF HOX 80%
PDGFRα
αSMA
GAPDH
— 250
— 150
— 50
— 37

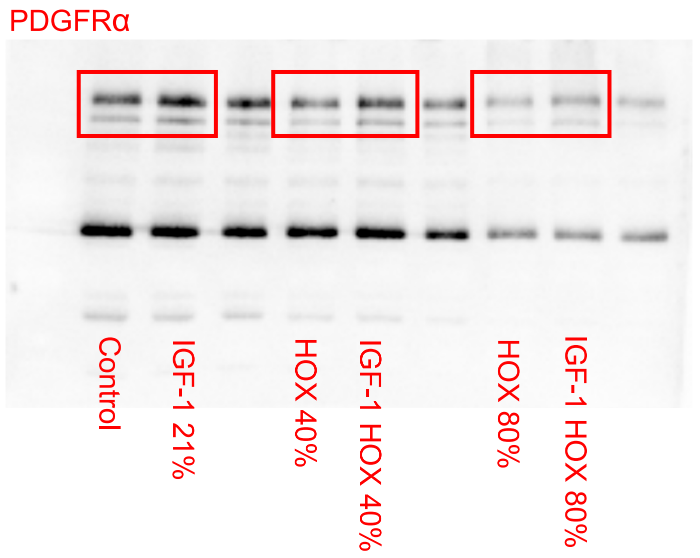
PDGFRα
Control
IGF-1 21%
HOX 40%
IGF-1 HOX 40%
HOX 80%
IGF-1 HOX 80%

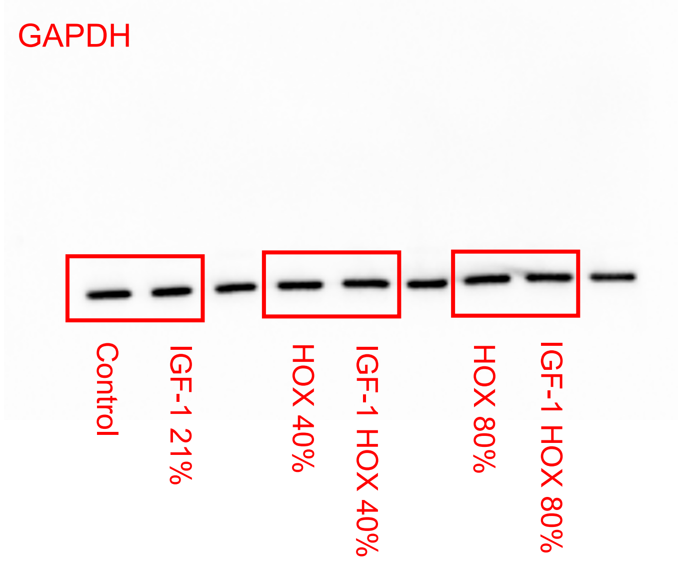
GAPDH
Control
IGF-1 21%
HOX 40%
IGF-1 HOX 40%
HOX 80%
IGF-1 HOX 80%

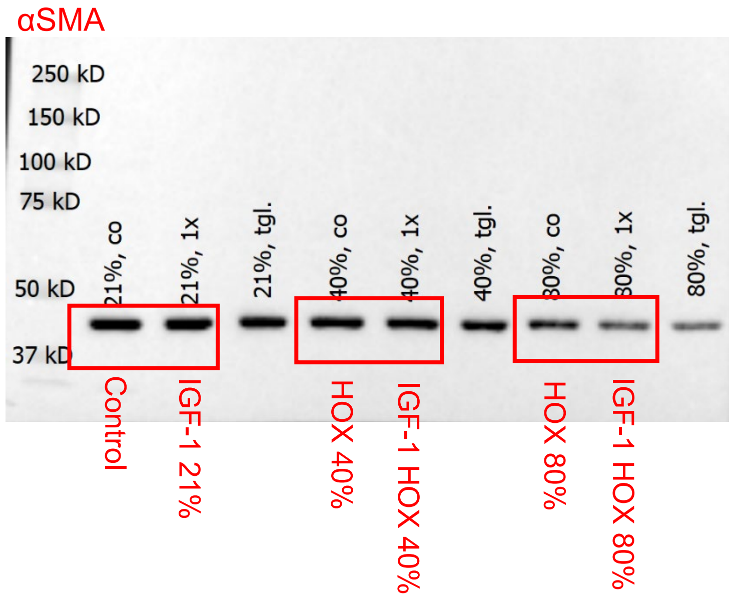
αSMA
250 kD
150 kD
100 kD
75 kD
50 kD
37 kD
21%, co
21%, 1x
21%, tgl.
40%, co
40%, 1x
40%, tgl.
80%, co
80%, 1x
80%, tgl.
Control
IGF-1 21%
HOX 40%
IGF-1 HOX 40%
HOX 80%
IGF-1 HOX 80%

Figure 3D

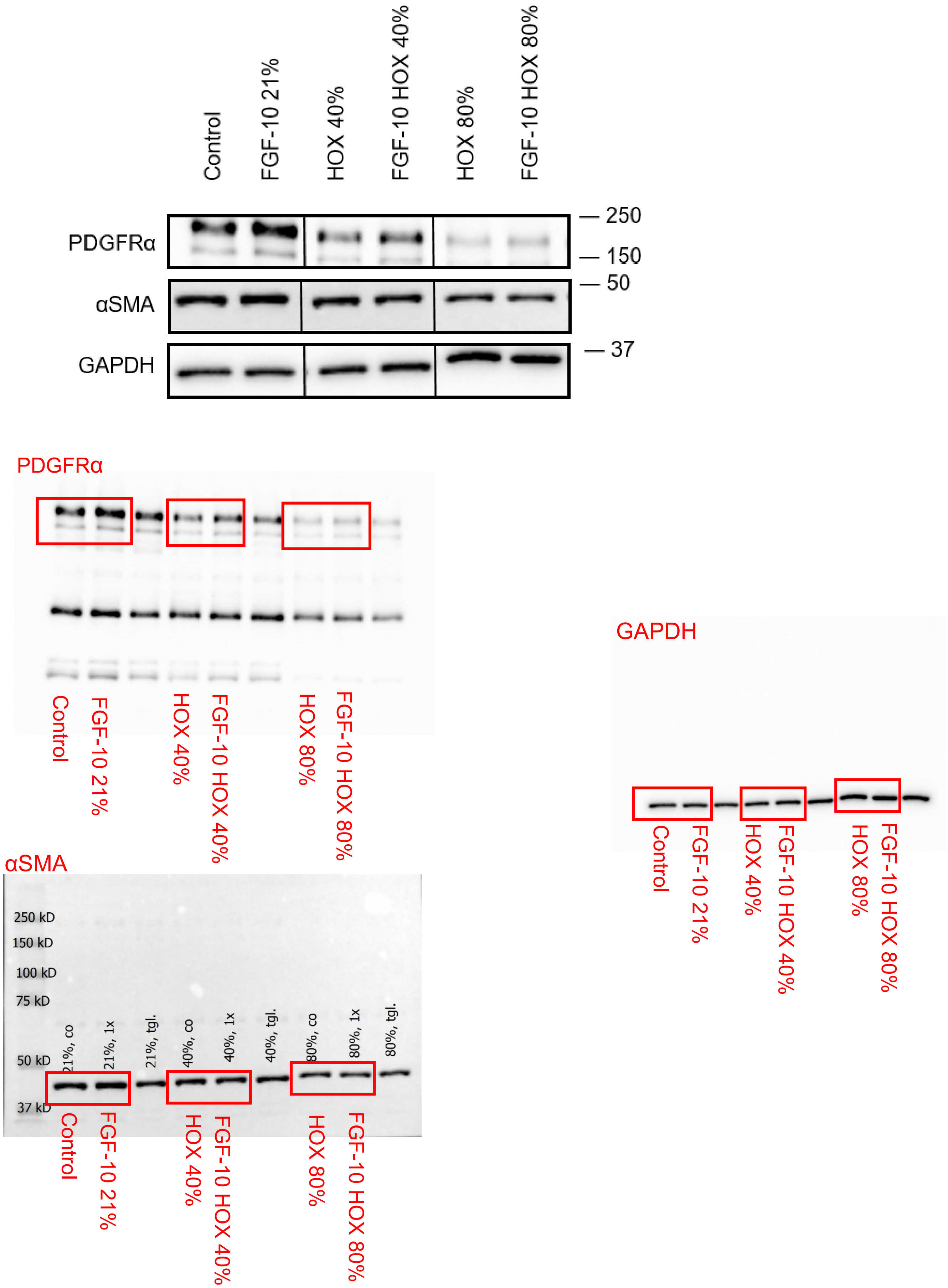

Control
FGF-10 21%
HOX 40%
FGF-10 HOX 40%
HOX 80%
FGF-10 HOX 80%
PDGFRα
αSMA
GAPDH
— 250
— 150
— 50
— 37
PDGFRα
Control
FGF-10 21%
HOX 40%
FGF-10 HOX 40%
HOX 80%
FGF-10 HOX 80%
αSMA
250 kD
150 kD
100 kD
75 kD
50 kD
37 kD
21%, co
21%, 1x
21%, tgl.
40%, co
40%, 1x
40%, tgl.
80%, co
80%, 1x
80%, tgl.
Control
FGF-10 21%
HOX 40%
FGF-10 HOX 40%
HOX 80%
FGF-10 HOX 80%
GAPDH
Control
FGF-10 21%
HOX 40%
FGF-10 HOX 40%
HOX 80%
FGF-10 HOX 80%

Figure 3E

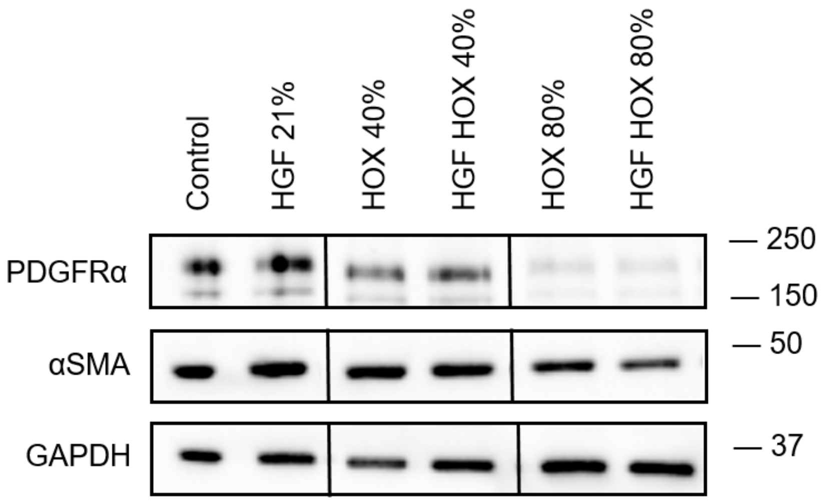

Control
HGF 21%
HOX 40%
HGF HOX 40%
HOX 80%
HGF HOX 80%
PDGFRα
αSMA
GAPDH
250
150
50
37

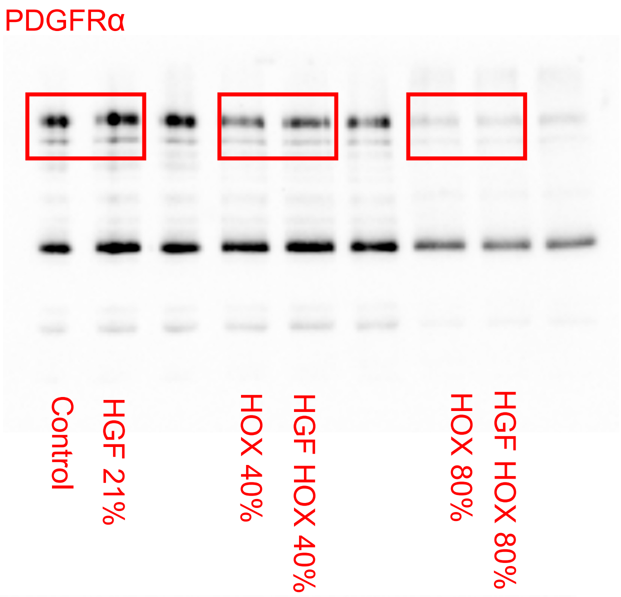

PDGFRα
Control
HGF 21%
HOX 40%
HGF HOX 40%
HOX 80%
HGF HOX 80%

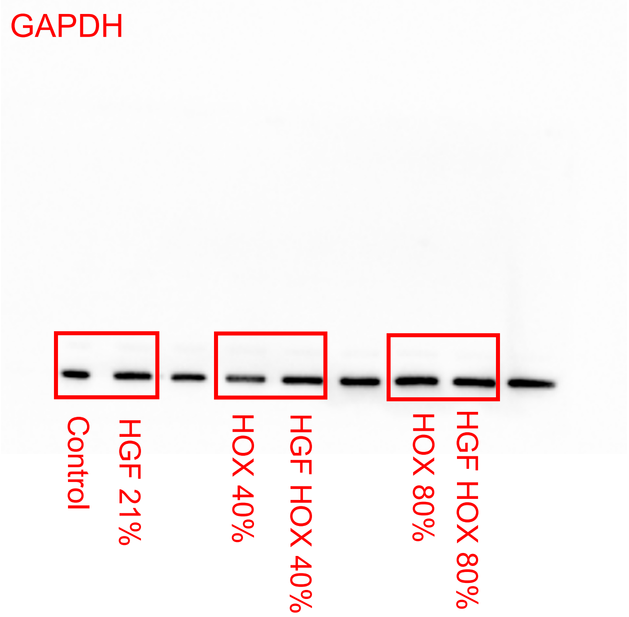

GAPDH
Control
HGF 21%
HOX 40%
HGF HOX 40%
HOX 80%
HGF HOX 80%

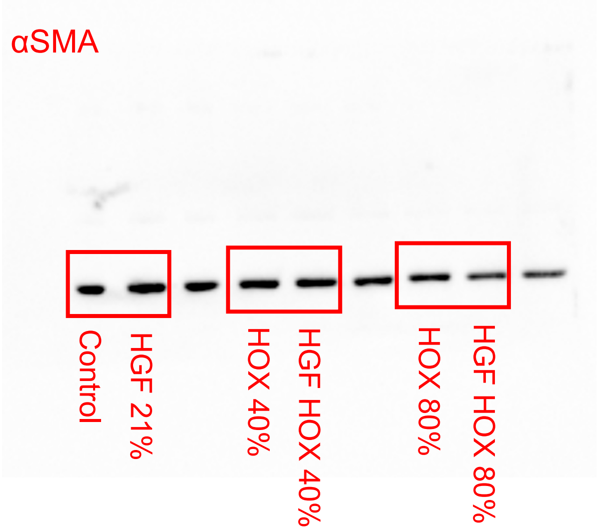

αSMA
Control
HGF 21%
HOX 40%
HGF HOX 40%
HOX 80%
HGF HOX 80%

Figure 3F

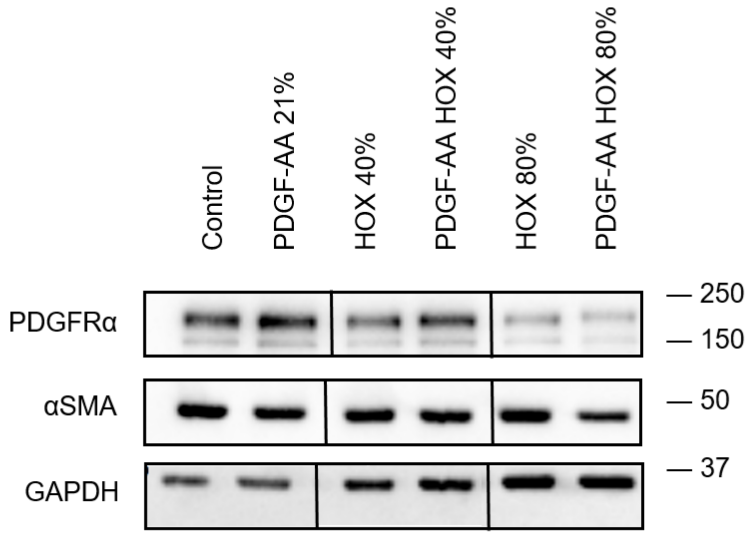
Control
PDGF-AA 21%
HOX 40%
PDGF-AA HOX 40%
HOX 80%
PDGF-AA HOX 80%
PDGFRα
αSMA
GAPDH
— 250
— 150
— 50
— 37

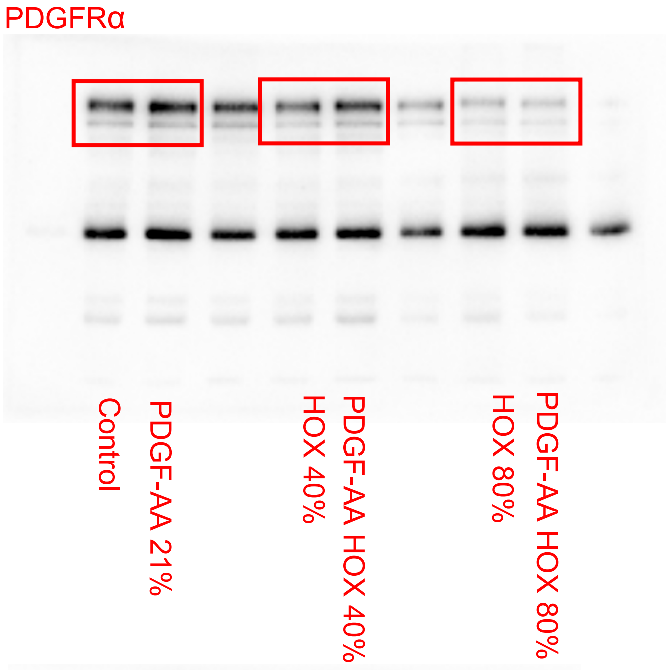
PDGFRα
Control
PDGF-AA 21%
HOX 40%
PDGF-AA HOX 40%
HOX 80%
PDGF-AA HOX 80%

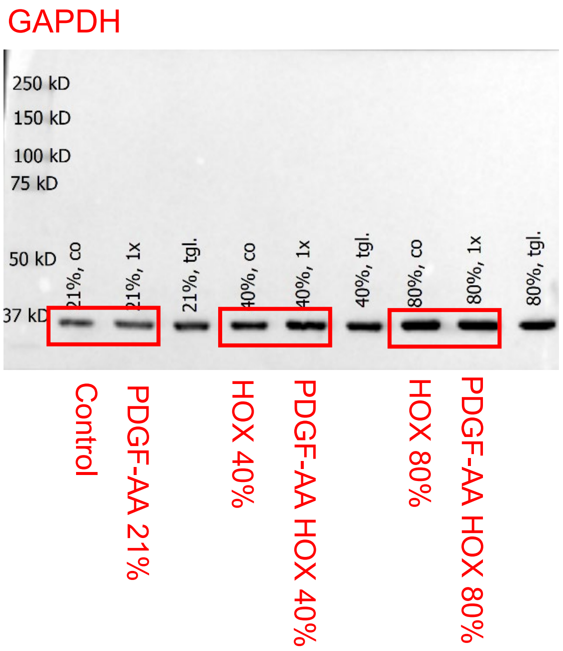
GAPDH
250 kD
150 kD
100 kD
75 kD
50 kD
37 kD
21%, co
21%, 1x
21%, tgl.
40%, co
40%, 1x
40%, tgl.
80%, co
80%, 1x
80%, tgl.
Control
PDGF-AA 21%
HOX 40%
PDGF-AA HOX 40%
HOX 80%
PDGF-AA HOX 80%

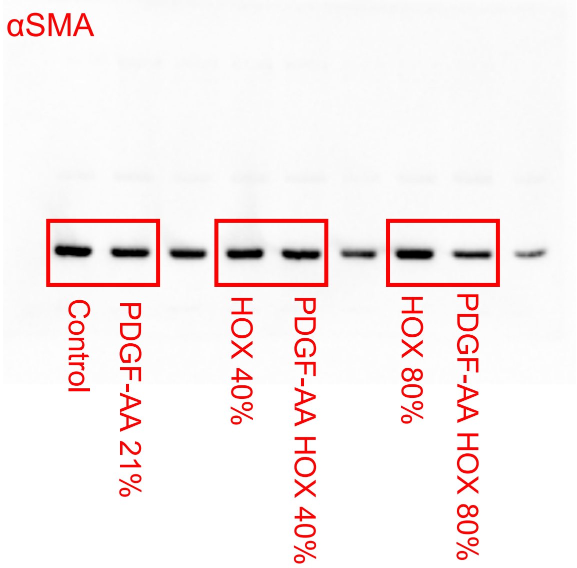
αSMA
Control
PDGF-AA 21%
HOX 40%
PDGF-AA HOX 40%
HOX 80%
PDGF-AA HOX 80%

Figure 4E

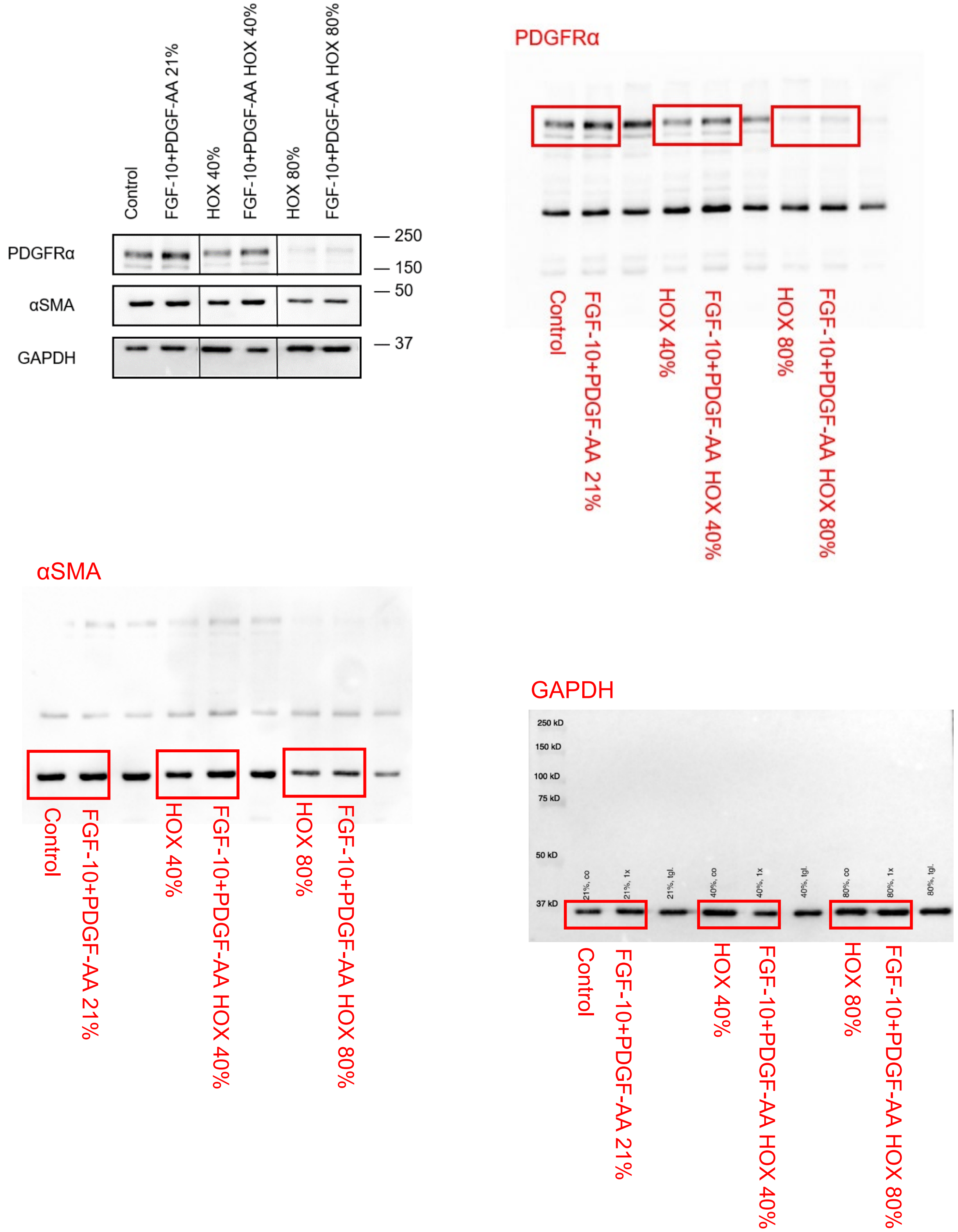
Control
FGF-10+PDGF-AA 21%
HOX 40%
FGF-10+PDGF-AA HOX 40%
HOX 80%
FGF-10+PDGF-AA HOX 80%
PDGFRα
αSMA
GAPDH
— 250
— 150
— 50
— 37
PDGFRα
Control
FGF-10+PDGF-AA 21%
HOX 40%
FGF-10+PDGF-AA HOX 40%
HOX 80%
FGF-10+PDGF-AA HOX 80%
αSMA
Control
FGF-10+PDGF-AA 21%
HOX 40%
FGF-10+PDGF-AA HOX 40%
HOX 80%
FGF-10+PDGF-AA HOX 80%
GAPDH
250 kD
150 kD
100 kD
75 kD
50 kD
37 kD
21%, co
21%, 1x
21%, tgl.
40%, co
40%, 1x
40%, tgl.
80%, co
80%, 1x
80%, tgl.
Control
FGF-10+PDGF-AA 21%
HOX 40%
FGF-10+PDGF-AA HOX 40%
HOX 80%
FGF-10+PDGF-AA HOX 80%

Supplemental Figure S2: Daily preventive application of lung growth promoting cytokines while hyperoxia (HOX) exposure of lung resident mesenchymal stem cells (MSC) of preterm infants and cell growth.

A

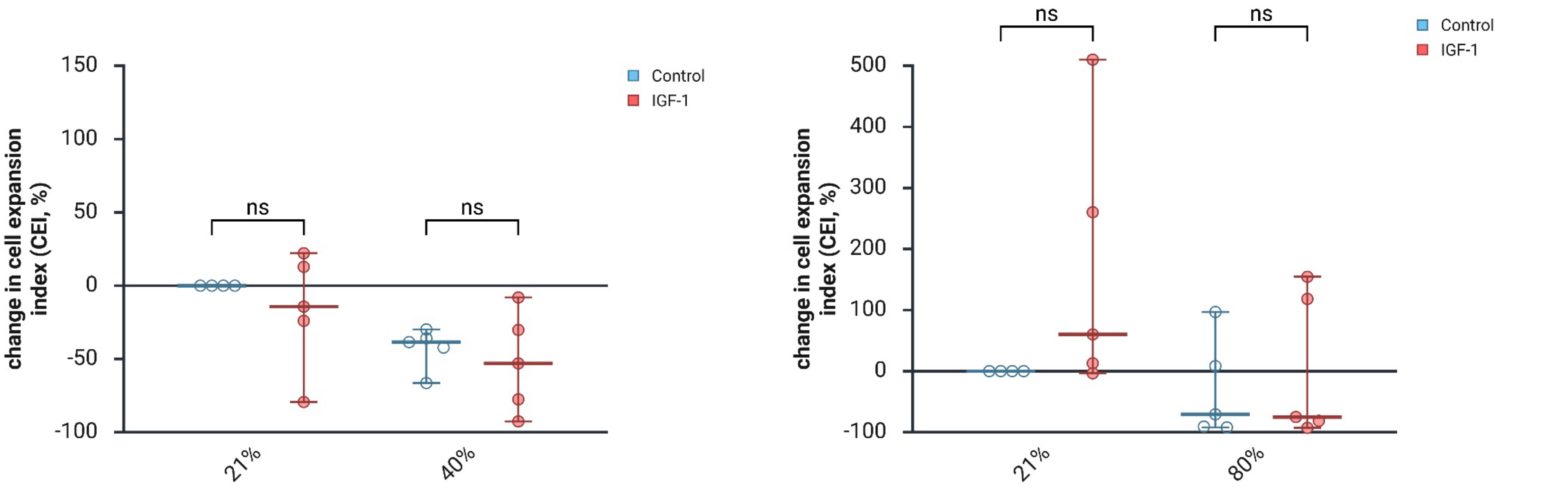

B

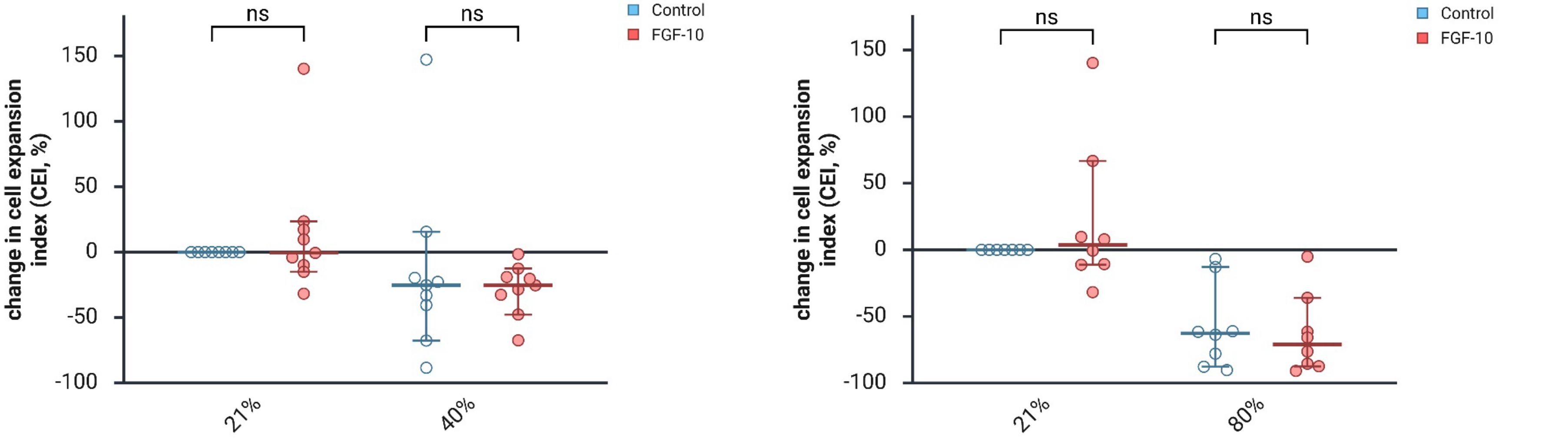

change in cell expansion index (CEI, %)
150
100
50
0
-50
-100
ns
ns
21%
40%
Control
FGF-10
change in cell expansion index (CEI, %)
150
100
50
0
-50
-100
ns
ns
21%
80%
Control
FGF-10

C

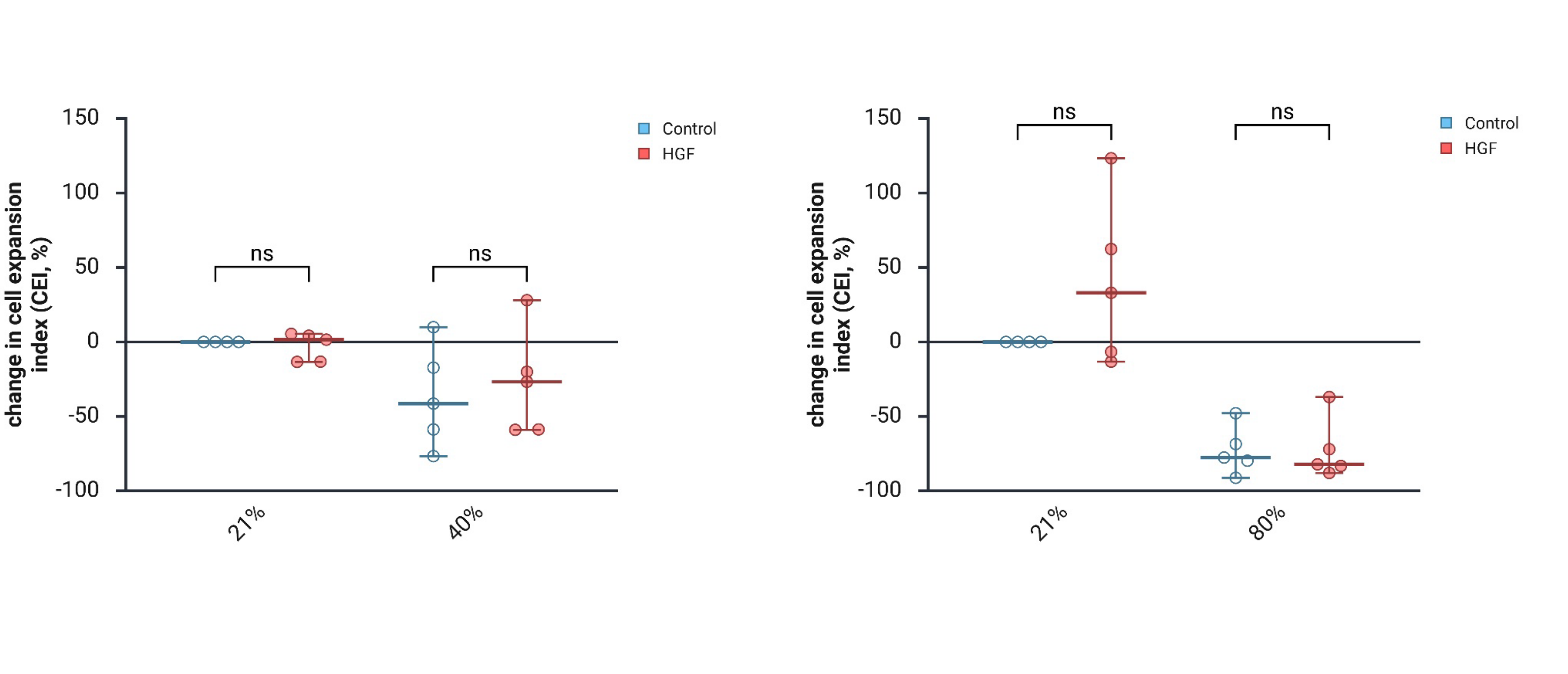

150
100
50
0
-50
-100
change in cell expansion index (CEI, %)
ns
ns
21%
40%
Control
HGF
150
100
50
0
-50
-100
change in cell expansion index (CEI, %)
ns
ns
21%
80%
Control
HGF

D

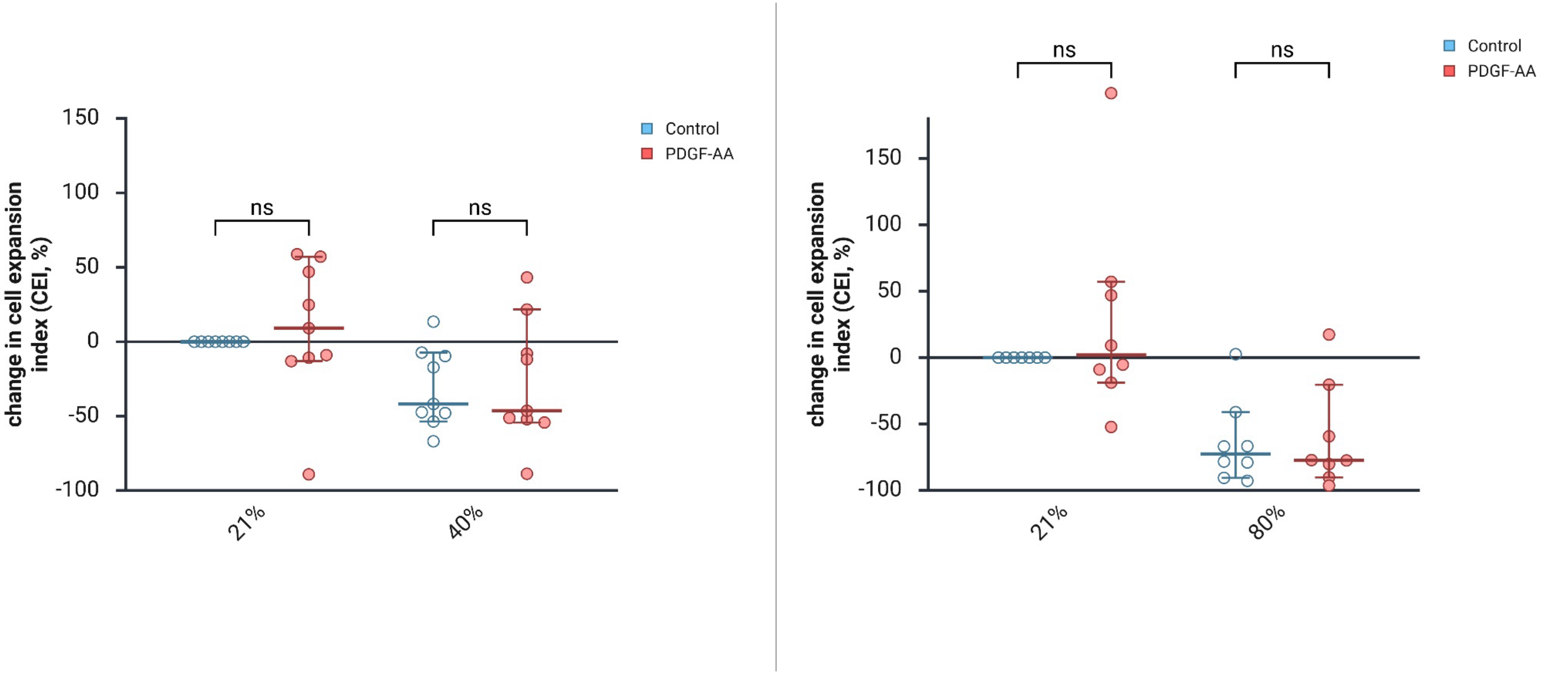
change in cell expansion index (CEI, %)
150
100
50
0
-50
-100
ns
ns
21%
40%
Control
PDGF-AA
change in cell expansion index (CEI, %)
150
100
50
0
-50
-100
ns
ns
21%
80%
Control
PDGF-AA

Supplemental Figure S3: Daily preventive application of lung growth promoting cytokines before hyperoxia (HOX) exposure of lung resident mesenchymal stem cells (MSC) of preterm infants and cell death induction.

A

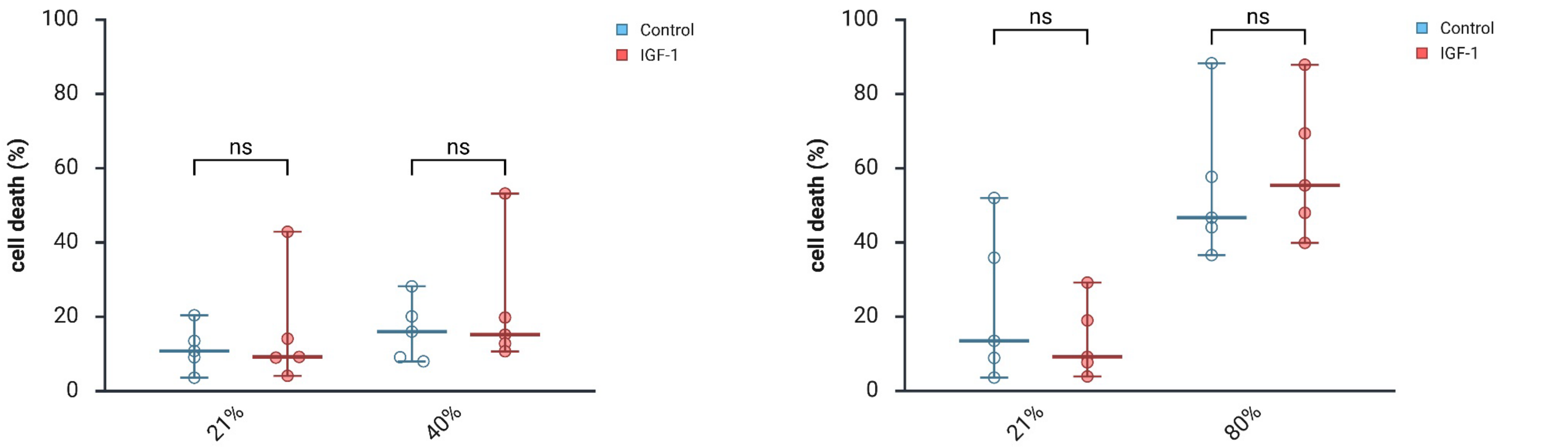

B

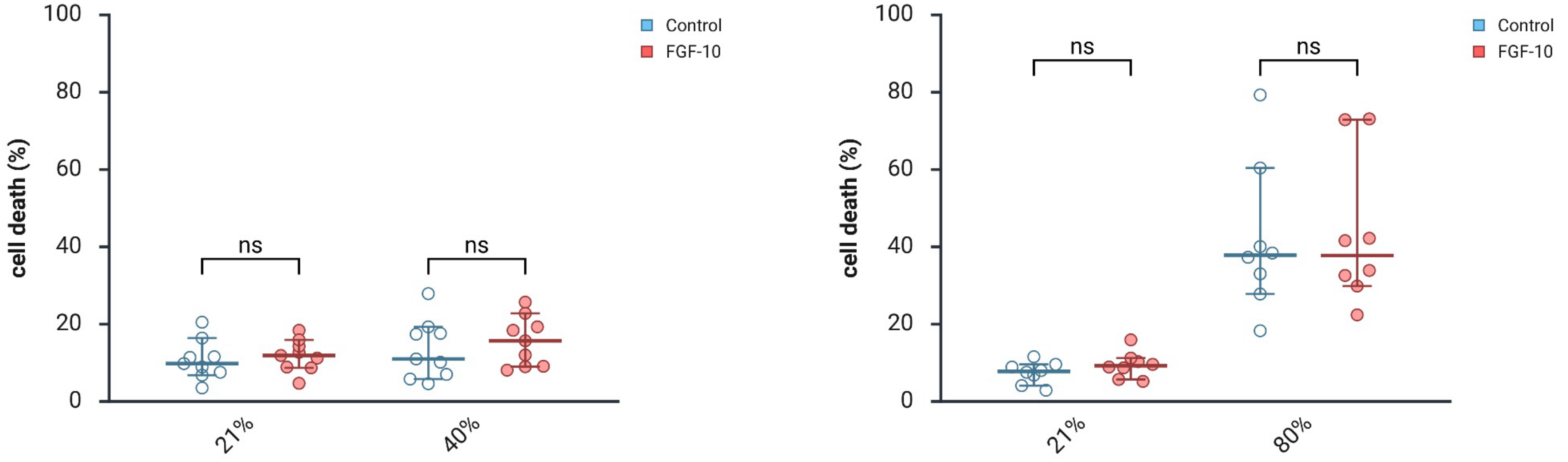
100
80
60
40
20
0
cell death (%)
ns
ns
21%
40%
Control
FGF-10
100
80
60
40
20
0
cell death (%)
ns
ns
21%
80%
Control
FGF-10

C

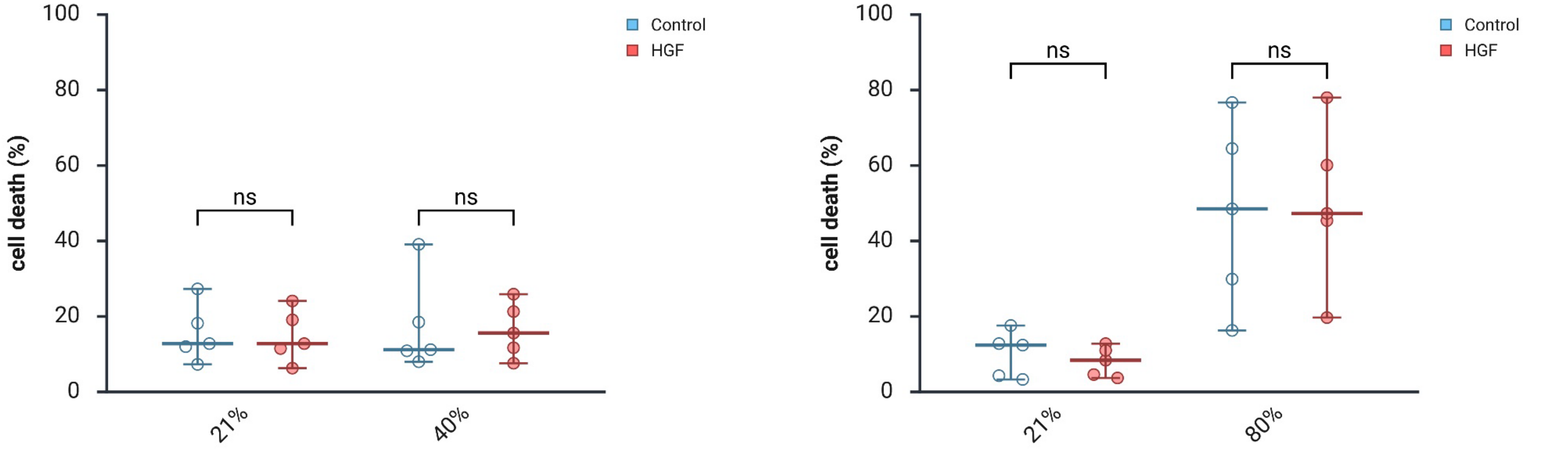

100
80
60
40
20
0
cell death (%)
ns
ns
21%
40%
Control
HGF
100
80
60
40
20
0
cell death (%)
ns
ns
21%
80%
Control
HGF

D

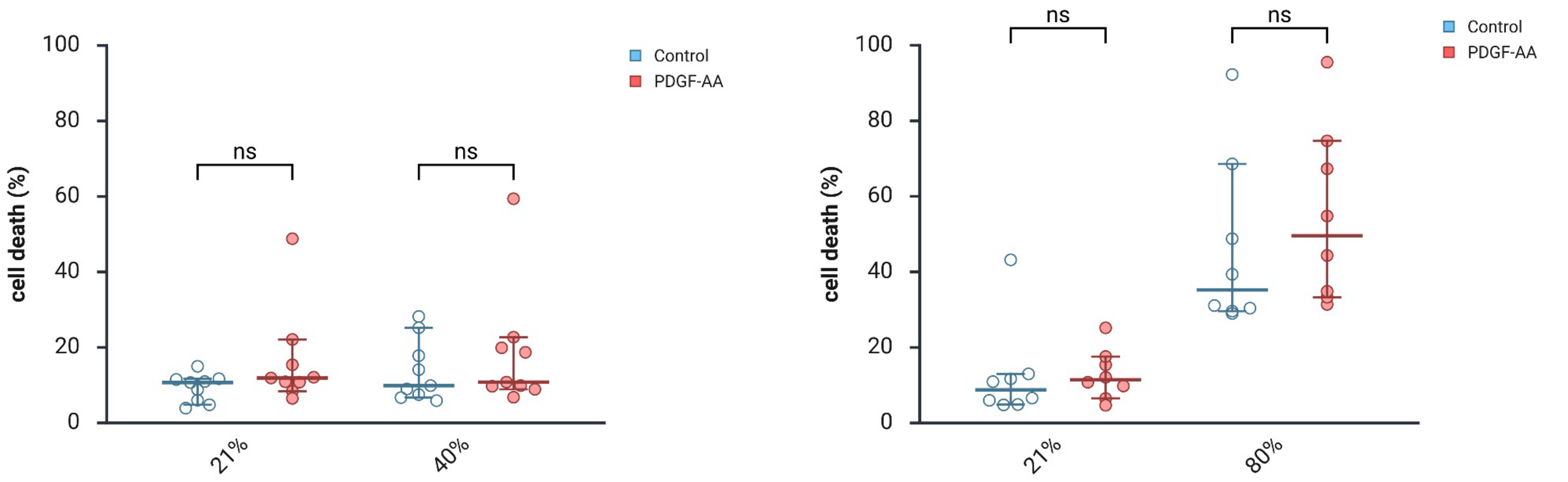

100
80
60
40
20
0
cell death (%)
ns
ns
21%
40%
Control
PDGF-AA
100
80
60
40
20
0
cell death (%)
ns
ns
21%
80%
Control
PDGF-AA

Supplemental Figure S4: Daily application of lung growth promoting cytokines and effect on hyperoxia mediated changes in the expression of lung resident MSC marker proteins.

A

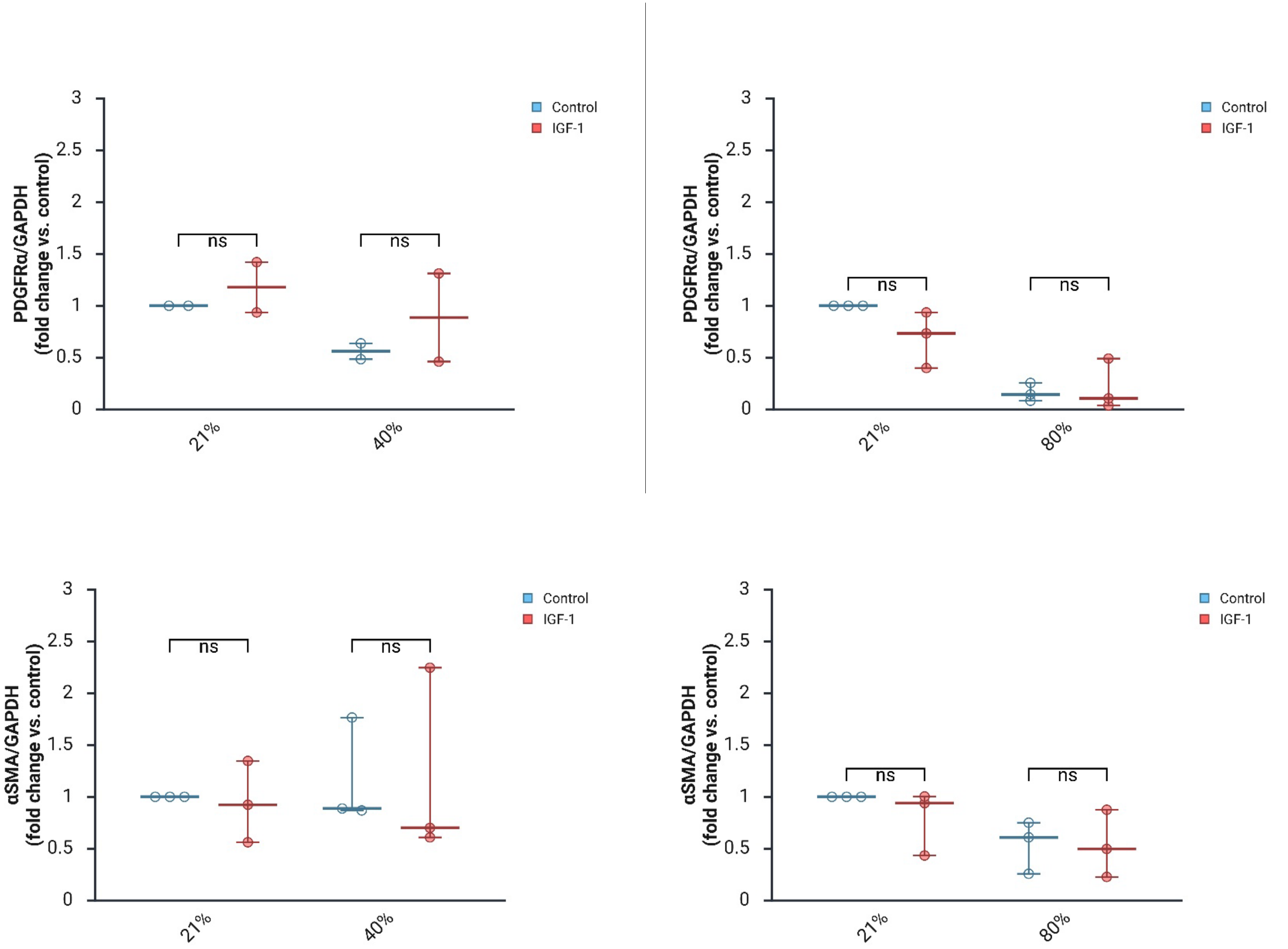

B

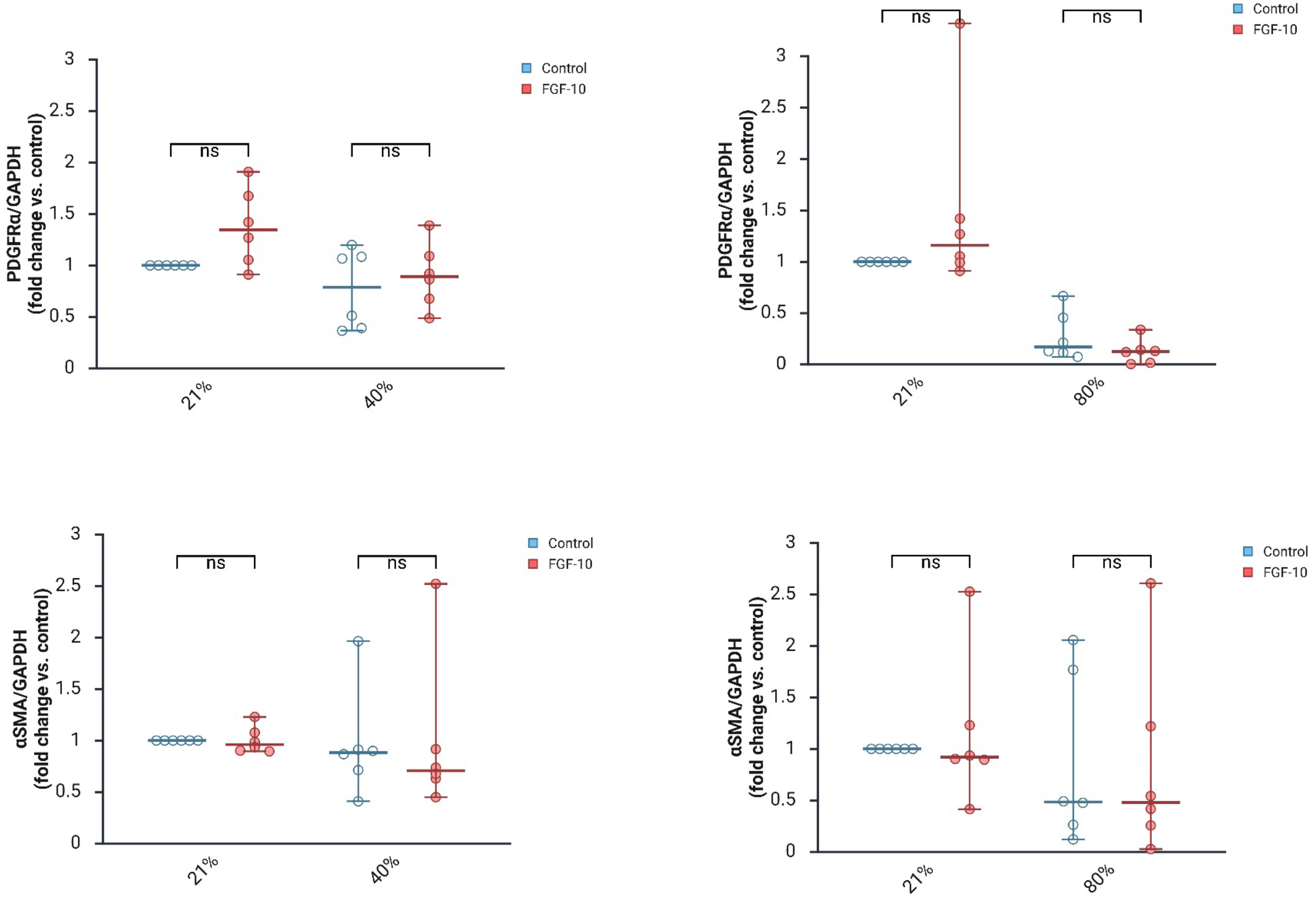
PDGFRα/GAPDH (fold change vs. control)
ns
ns
Control
FGF-10
0
0.5
1
1.5
2
2.5
3
21%
40%
80%
αSMA/GAPDH (fold change vs. control)

C

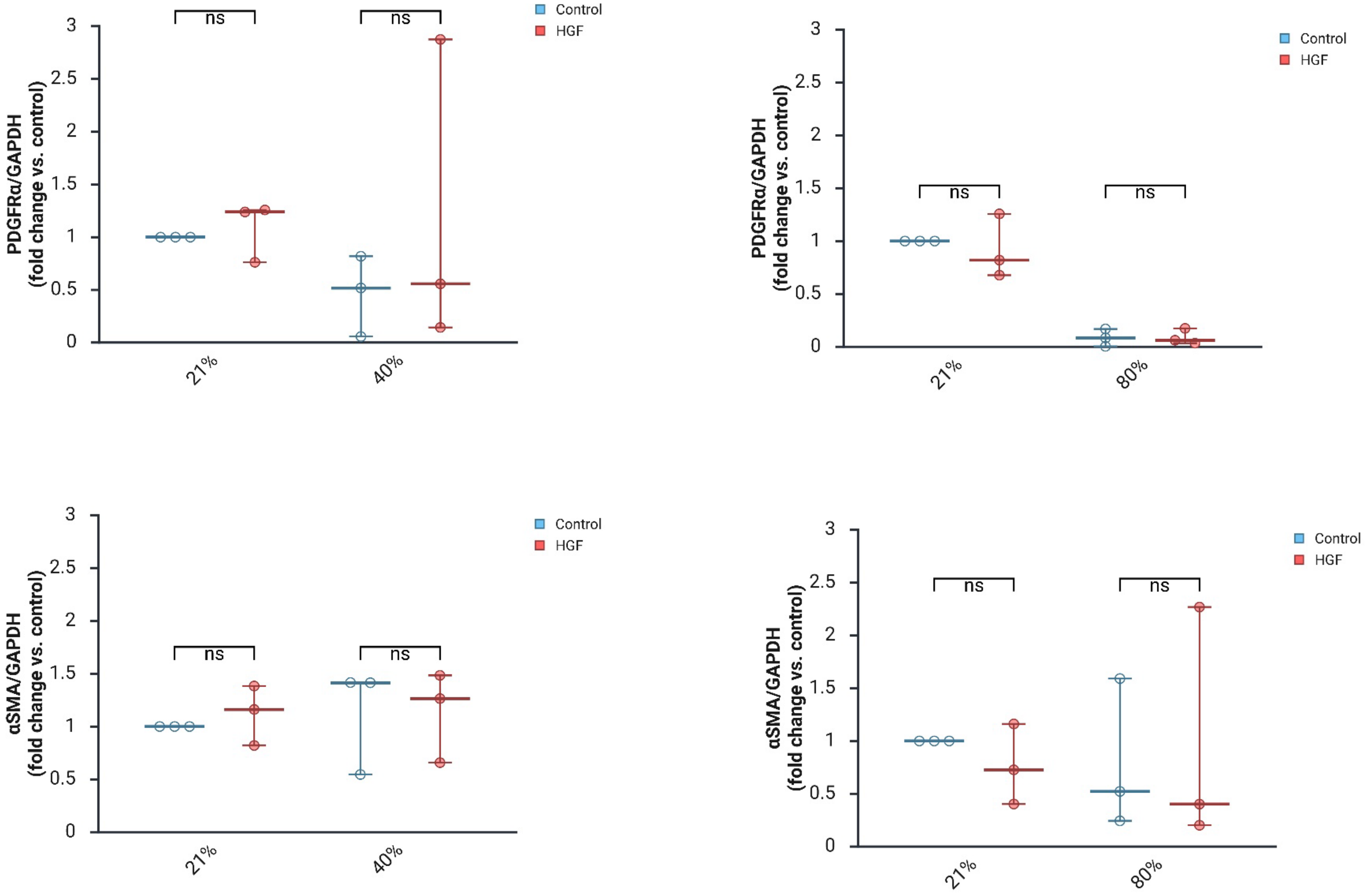

PDGFRα/GAPDH
(fold change vs. control)
ns
ns
Control
HGF
3
2.5
2
1.5
1
0.5
0
21%
40%
PDGFRα/GAPDH
(fold change vs. control)
ns
ns
Control
HGF
21%
80%
αSMA/GAPDH
(fold change vs. control)
ns
ns
Control
HGF
21%
40%
αSMA/GAPDH
(fold change vs. control)
ns
ns
Control
HGF
21%
80%

D

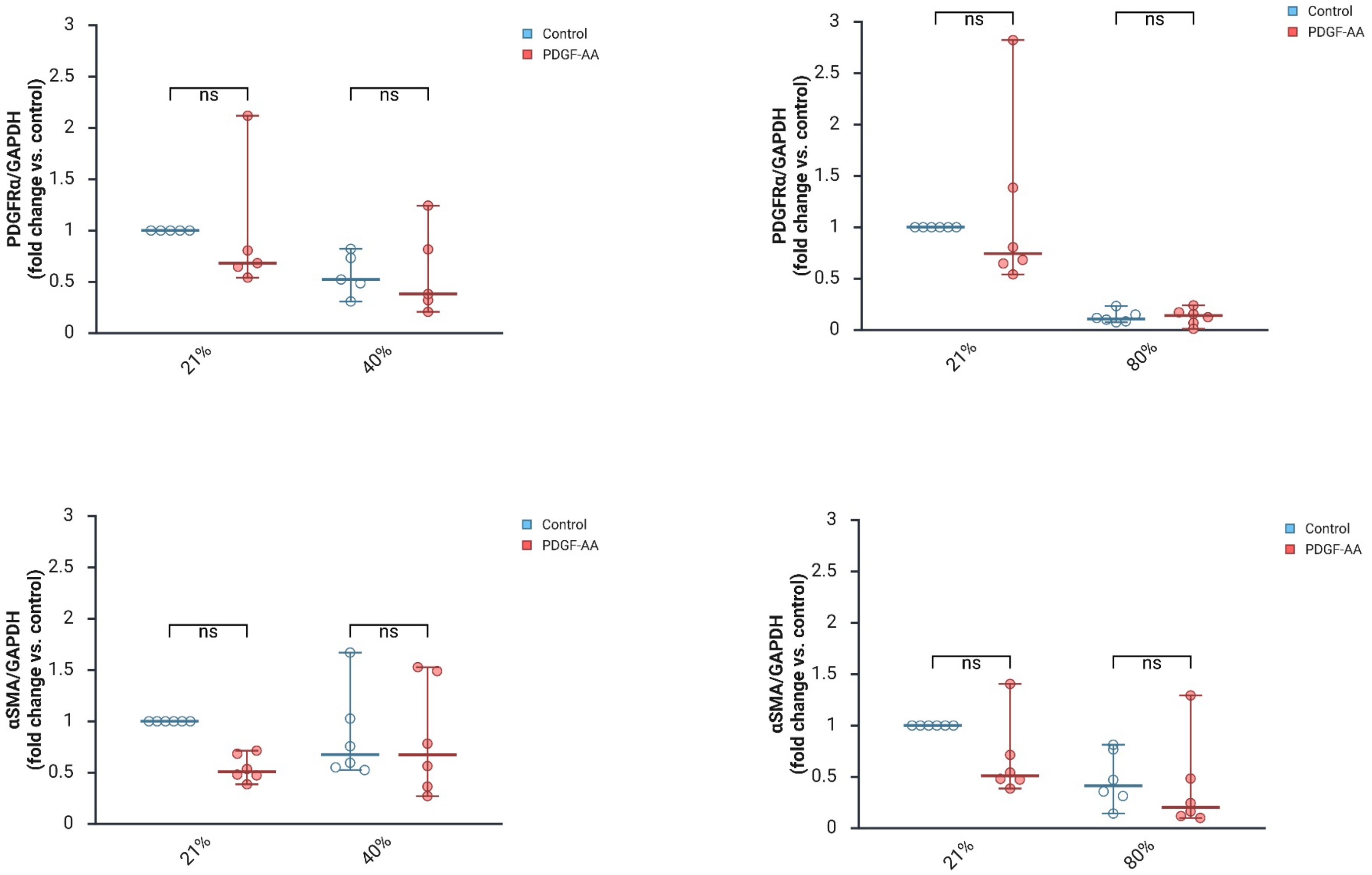

PDGFRα/GAPDH (fold change vs. control)
ns
ns
21%
40%
Control
PDGF-AA
PDGFRα/GAPDH (fold change vs. control)
ns
ns
21%
80%
Control
PDGF-AA
αSMA/GAPDH (fold change vs. control)
ns
ns
21%
40%
Control
PDGF-AA
αSMA/GAPDH (fold change vs. control)
ns
ns
21%
80%
Control
PDGF-AA

Supplemental Figure 5: Daily Combinatorial pretreatment with FGF-10 and PDGF-AA

A

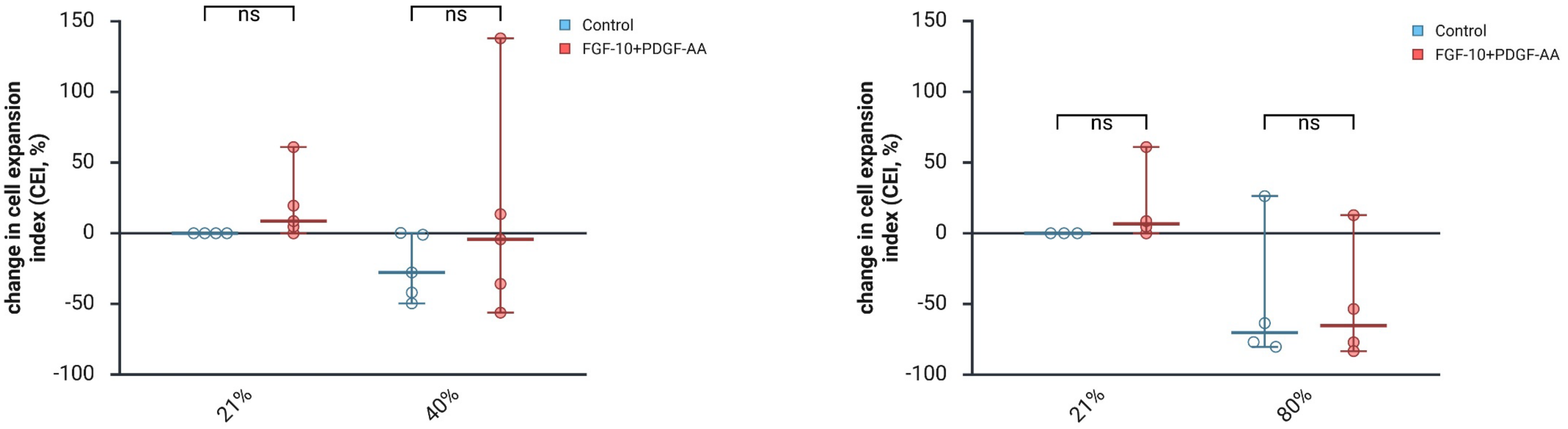

B

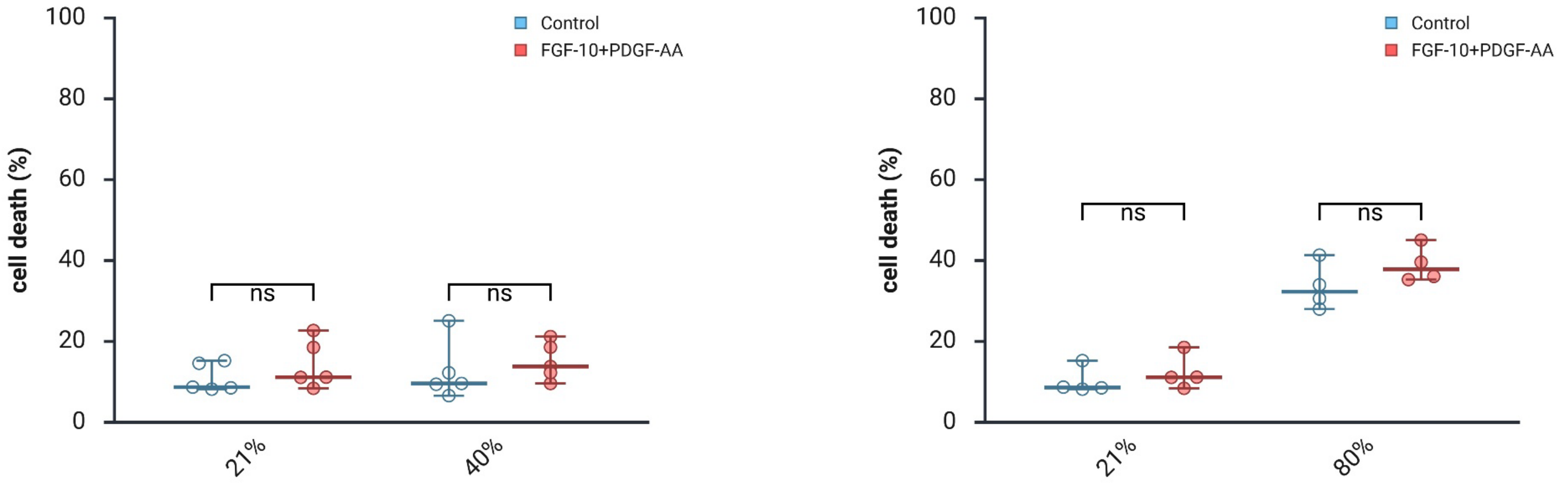
100
80
60
40
20
0
cell death (%)
Control
FGF-10+PDGF-AA
ns
ns
21%
40%
100
80
60
40
20
0
cell death (%)
Control
FGF-10+PDGF-AA
ns
ns
21%
80%

C

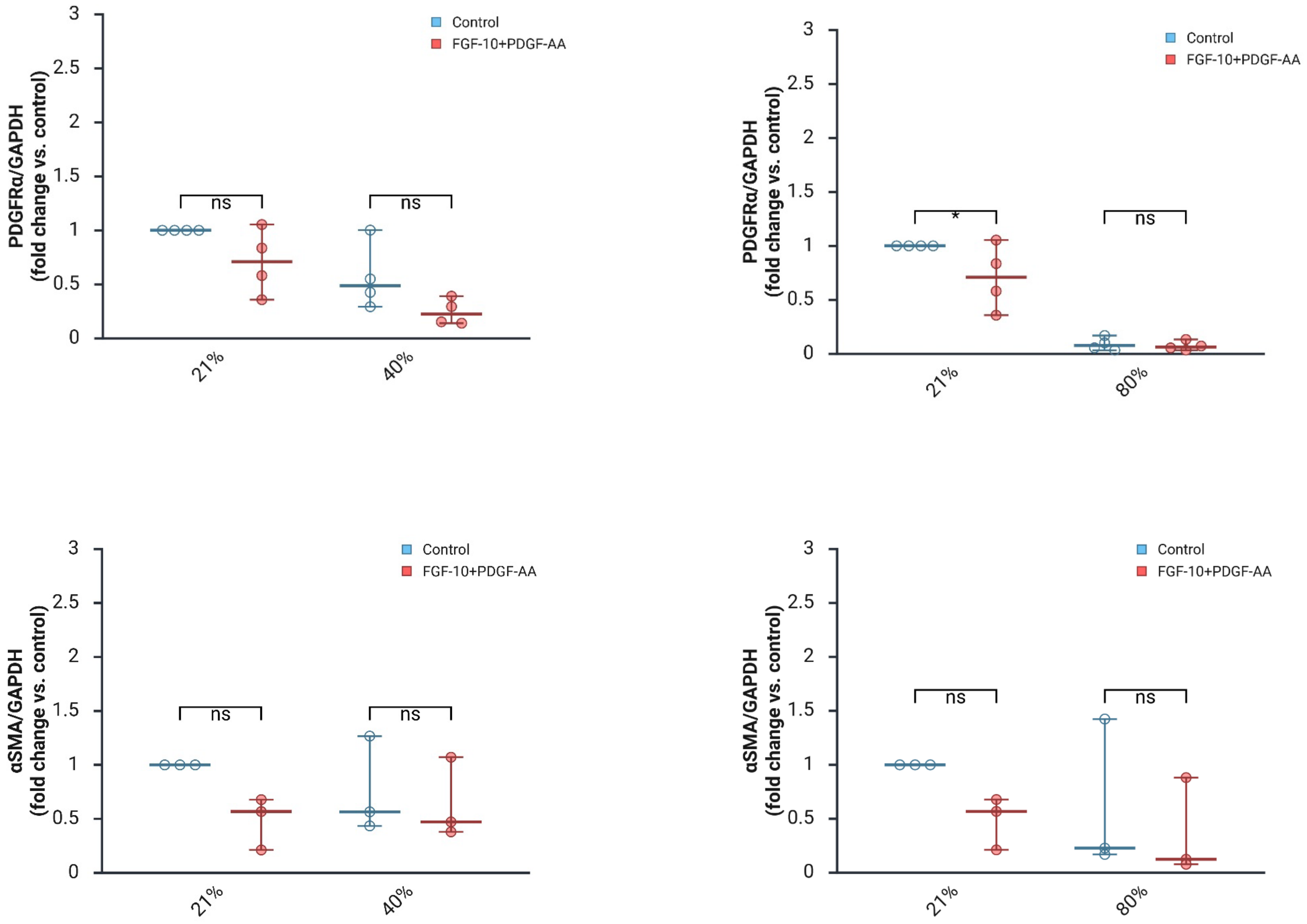
PDGFRα/GAPDH (fold change vs. control)
Control
FGF-10+PDGF-AA
ns
ns
21%
40%
3
2.5
2
1.5
1
0.5
0
PDGFRα/GAPDH (fold change vs. control)
Control
FGF-10+PDGF-AA
*
ns
21%
80%
αSMA/GAPDH (fold change vs. control)
Control
FGF-10+PDGF-AA
ns
ns
21%
40%
αSMA/GAPDH (fold change vs. control)
Control
FGF-10+PDGF-AA
ns
ns
21%
80%
